# Generation of Dynamic Combinatorial Libraries Using Hydrazone‐Functionalized Surface Mimetics

**DOI:** 10.1002/ejoc.201800022

**Published:** 2018-04-25

**Authors:** Sarah H. Hewitt, Andrew J. Wilson

**Affiliations:** ^1^ School of Chemistry University of Leeds Woodhouse Lane 9JT Leeds LS2 UK; ^2^ Astbury Centre for Structural Molecular Biology University of Leeds Woodhouse Lane 9JT Leeds LS2 UK

**Keywords:** Dynamic combinatorial chemistry, Hydrazones, Protein surface mimetics, Porphyrins

## Abstract

Dynamic combinatorial chemistry (DCC) represents an approach, whereby traditional supramolecular scaffolds used for protein surface recognition might be exploited to achieve selective high affinity target recognition. Synthesis, in situ screening and amplification under selection pressure allows the generation of ligands, which bear different moieties capable of making multivalent non‐covalent interactions with target proteins. Generic tetracarboxyphenyl porphyrin scaffolds bearing four hydrazide moieties have been used to form dynamic combinatorial libraries (DCLs) using aniline‐catalyzed reversible hydrazone exchange reactions, in 10 % DMSO, 5 mm NH_4_OAc, at pH 6.75. High resolution mass spectrometry (HRMS) was used to monitor library composition and establish conditions under which equilibria were established.

## Introduction

A central goal in supramolecular chemical biology^1^ is to generate ligands capable of solvent exposed protein‐surface recognition for the purpose of orthosteric protein–protein interaction (PPI) inhibition.^2^, ^3^, ^4^ Inhibition of PPIs is considered challenging in view of the fact that such interactions involve extended and less well‐defined surfaces than the “lock‐and‐key”‐like interfaces that have historically proven to be tractable targets for drug‐discovery.^4^ Several supramolecular scaffolds have been explored as templates upon which to elaborate ligands for selective and high affinity protein‐surface binding,^5^ including: porphyrins,^6^, ^7^, ^8^, ^9^ calixarenes,^9^, ^10^, ^11^, ^12^, ^13^ cucurbiturils,^14^, ^15^ molecular clips,^16^, ^17^ ruthenium(II) tris‐chelates^18^, ^19^, ^20^, ^21^, ^22^, ^23^ and other ligands.^24^, ^25^, ^26^, ^27^ These function by projecting multiple binding groups from the scaffold so as to cover a large surface area and make multivalent contacts with the protein surface.^5^ A limitation in the use of such scaffolds is that they are typically highly symmetrical, with identical binding groups being projected from the periphery, posing a challenge in terms of achieving adequate selectivity.^5^, ^6^ A potential solution to this challenge would be to exploit dynamic covalent chemistry (DCC) to synthesize dynamic combinatorial libraries (DCLs)^28^, ^29^, ^30^, ^31^, ^32^ of protein‐surface mimetics^33^, ^34^ bearing different functional motifs at the periphery. Such DCLs comprise many different receptors that interconvert at equilibrium. The addition of a protein target places the DCL under selection pressure, resulting in enrichment of protein‐binding ligands. This could allow the coupling of synthesis with initial screening, whereby the protein should template and amplify synthesis of the highest affinity ligand(s) from the DCL.^31^ Hydrazone exchange chemistry has been used in a range of DCC studies including host–guest systems,^35^ interlocked^36^ architectures, polymers^37^, ^38^ and nanoparticles:^39^ it is an attractive reversible covalent bond forming reaction for DCC in water because the resultant hydrazone bond is stable in water, but its use has been limited because the exchange reaction typically operates at acidic pH. Both Dawson and co‐workers and Greaney and co‐workers elaborated methods to render the use of hydrazone exchange more useful under biologically relevant conditions by introducing aniline as a nucleophilic catalyst, to facilitate the reaction at pH 6.2.^40^, ^41^ Several alternative catalysts have subsequently been introduced.^42^, ^43^ Despite this progress, the exploitation of hydrazone‐exchange in aqueous solvent on supramolecular scaffolds used for protein surface recognition has not been reported, although studies on related α‐helix mimetics have been carried out in DMSO.^44^ In the current manuscript, we describe efforts focused on the underpinning synthesis and analytical chemistry needed to exploit hydrazone‐functionalized porphyrins in DCLs of potential protein surface mimetics. Reliable conditions for hydrazone exchange are described (5 mm aniline, 10 % DMSO, 5 mm NH_4_OAc, pH 6.75, ca. 12 h) together with high resolution mass spectrometry (HRMS) analyses, which establish that DCLs with 3 different aldehyde components can be readily analysed and all 15 products identified (based on 4 hydrazones per porphyrin).

## Results and Discussion

**Scheme 1:**
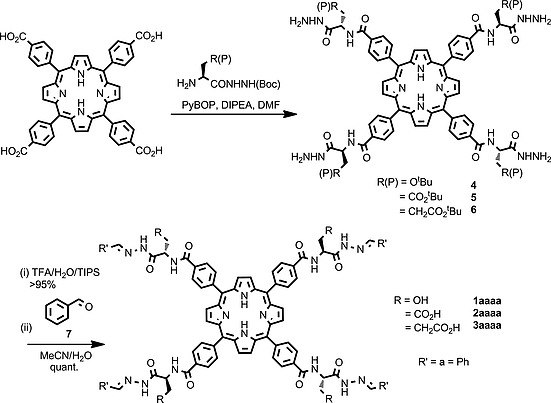
Synthesis of hydazone‐functionalized porphyrins **1**–**3aaaa**.

A tetraphenylporphyrin scaffold was chosen as a multivalent hydrazone scaffold. This choice was motivated by the ready accessibility of commercial porphyrins with a sufficient number of points at which a hydrazide linker could be introduced, and the established utility of porphyrins in protein surface recognition.^6^, ^7^, ^8^, ^9^ Three different hydrazone porphyrins **1**–**3aaaa** (Scheme 1) were exploited in exchange studies; in each case a different amino acid spacer was used (**1** = Ser, **2** = Asp, **3** = Glu) between the porphyrin scaffold and exchangeable hydrazone bond. The use of different amino acids was intended to allow improved scaffold solubility and to investigate the role of functionality proximal to the hydrazone linker in terms of influencing exchange rate.

### Synthesis and Scaffold Design of Hydrazone‐Functionalized Porphyrin Scaffolds

Attempts to synthesise tetracarboxyhydrzidophenylporphyrin resulted in analytically intractable products (see Supporting information). Subsequent efforts centred on the introduction of an amino acid spacer between the porphyrin scaffold and the labile hydrazone linker. Initial synthesis using a glycine spacer resulted in formation of an insoluble product (see Supporting information). Therefore amino acid spacers containing groups to aid scaffold solubility, serine, aspartic acid and glutamic acid, were used. The introduction of the acid functionalities was also envisioned to aid the hydrazone exchange chemistry necessary during the DCL generation, through intramolecular proton transfer effects as has been observed in recent work from the Kool group.^45^ Successful syntheses of the target porphyrins **1**–**3aaaa** were achieved by direct coupling of protected amino acid hydrazides with tetracarboxyphenylporphyrin, using PyBOP, to generate protected hydrazide porphyrins **4**–**6**. Hydrazone scaffolds **1**–**3aaaa** ready for DCC studies bearing a simple phenyl hydrazone at each exchangeable bond were obtained from the corresponding protected hydrazides **4**–**6** by TFA deprotection followed by reaction with benzaldehyde **7**.

### Analysis of Hydrazone Exchange on Functionalized Porphyrins Using High Resolution Mass Spectrometry

**Figure 1:**
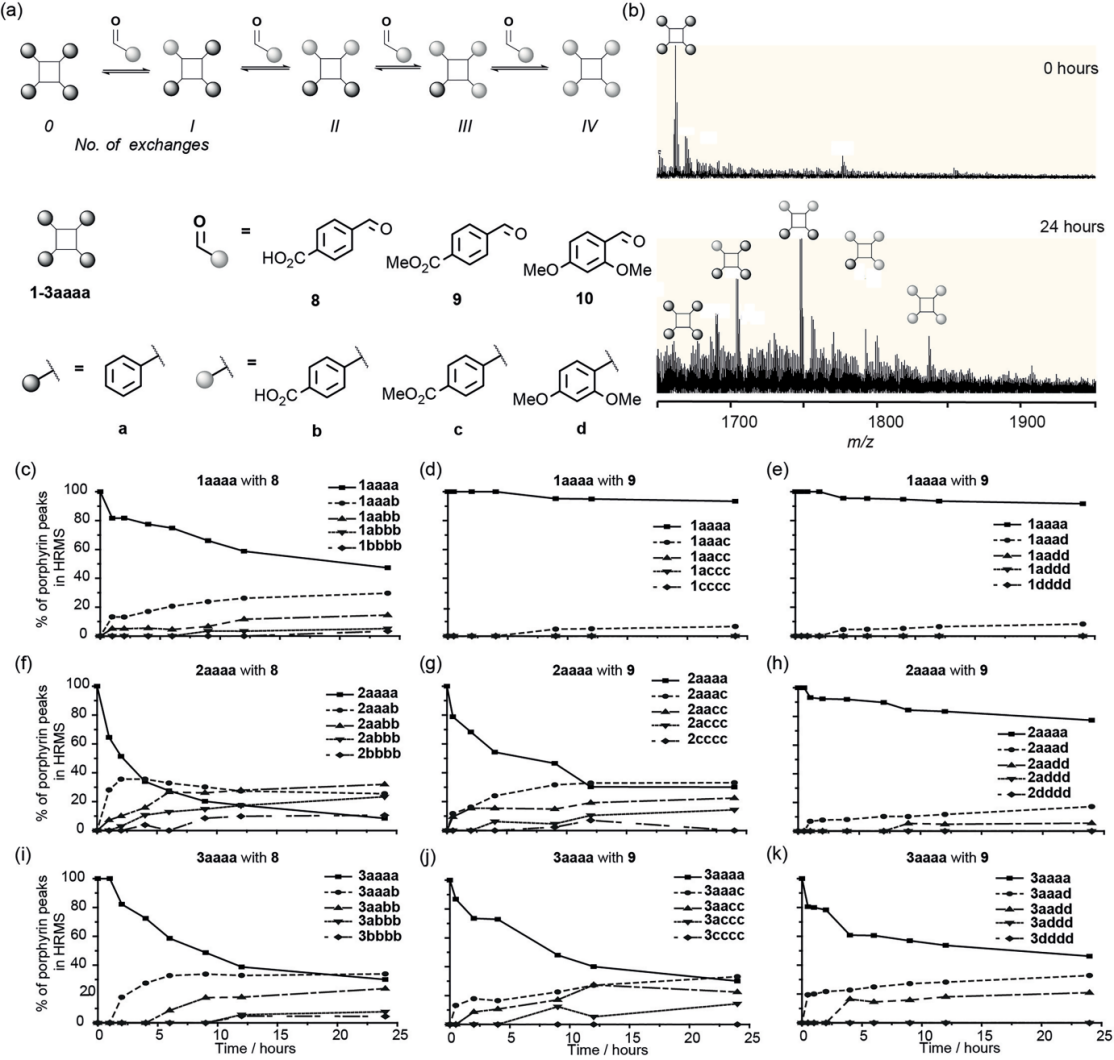
Initial hydrazone exchange time courses on porphyrin hydrazone scaffolds; (a) schematic depicting exchange process and structures; (b) exemplary HRMS showing hydrazone exchange on the porphyrin **1aaaa** with (aldehyde **8**). HRMS trace in porphyrin +1 region at start of incubation (top) and after 24 h incubation (bottom); (b)‐(k) relative speciation observed over 24 h (separate lines on the graphs correspond to different numbers of benzaldehyde moieties having exchanged for a new aldehyde); (c), (d) and (e) serine hydrazone porphyrin **1aaa**, (f), (g) and (h) aspartic acid hydrazone porphyrin **2aaaa**, (i), (j) and (k) glutamic acid hydrazone porphyrin **3aaaa**; incubated with 4‐carboxybenzaldehyde **8** (c), (f) and (i), 4‐methyl ester benzaldehyde **9** (d), (g) and (j) and 2,4‐dimethoxybenzaldehyde **10** (e), (h) and (k) for 24 h (conditions: 100 µm **1**–**3aaaa**, 5 mm **8**–**10**, 10 % DMSO, 5 mm NH_4_OAc, pH 6.75).

High resolution mass spectrometry (HRMS) was used to follow hydrazone exchange. Preliminary efforts to employ HPLC to monitor library distribution indicated that identification of gradients to separate closely related products would be challenging, and in the context of a screening workflow, HRMS represents a method to establish compound identity and qualitative changes in library composition in response to template. Hydrazone exchanges were performed with the three different porphyrin scaffolds **1**–**3aaaa**, with three different aldehydes: 4‐carboxybenzaldehyde **8**, 4‐methyl ester benzaldehyde **9** and 2,4‐dimethoxybenzaldehyde **10** (Figure 1a). Hydrazone exchanges were initially performed without a catalyst using 50 equivalents of aldehyde compared to the hydrazone porphyrin (12.5 equivalents per hydrazone). Hydrazone exchange was followed over time in order to establish: (i) if hydrazone exchange occurred, (ii) if equilibrium was reached and (iii) the timescale for the equilibrium to be established.

Considering one of these systems, (aspartic acid hydrazone porphyrin **2aaaa** with **8**), hydrazone exchange was observed within 24 h, and could be visualised by HRMS (Figure 1b), with clearly discernible mass peaks corresponding to each of the hydrazone exchange products. Additional peaks in the mass spectrum arise from sodiated and ammoniated species for each of the hydrazone exchanges, some N–N bond fragmentation during ionization was also observed. Considering only protonated peaks corresponding to each of the hydrazine exchange products, progression of hydrazone exchange could be monitored over time (Figure 1f). The initial species **2aaaa** decreased over time, one hydrazone exchange (**2aaab**) to **8**, at first increasing then decreasing. The products of successive hydrazone exchanges (**2aabb**, **2abbb** and **2bbbb**) then followed in succession.

Similar analyses were carried out for the porphyrin hydrazone scaffold **2aaaa** with aldehydes **9** and **10** (Figure 1g–h) and the remaining two porphyrin hydrazone scaffolds **1aaaa** and **3aaaa** with all the three different aldehydes (**8–10**) (Figure 1c–e and Figure 1i–k). These confirmed that all three porphyrin scaffolds might be suitable starting points for the elaboration of dynamic combinatorial protein surface mimetics, with hydrazone exchange occurring in a biologically relevant buffer, at ambient temperature over roughly 24 h. Different rates of hydrazone exchange were observed between the three porphyrin scaffolds and with the different aldehydes. Generally, the glutamic acid and aspartic acid hydrazone porphyrins **2aaaa** and **3aaaa** showed faster exchange rates than the serine hydrazone porphyrin **1aaaa**. This supports the hypothesis that the acid functionality may assist hydrazone exchange through intramolecular proton‐transfer effects.^45^ Similarly, the effect on hydrazone exchange kinetics of aromatic aldehydes bearing different substituents has been studied.^46^ Aldehydes **8** and **9** were also observed to exchange at a faster rate than **10**; this is likely due to the electron‐withdrawing nature of the *para*‐carbonyl activating the aldehyde/imine to nucleophilic attack, whereas the electron‐donating *ortho*‐ and *para*‐methoxy groups deactivate the aldehyde/imine to attack. However, in almost all cases the distribution of products still appeared to be changing and thus equilibrium was not reached after 24 h necessitating addition of a catalyst to increase the rate of reaction.

### Catalysis of Hydrazone Exchange on Functionalized Porphyrins

**Figure 2:**
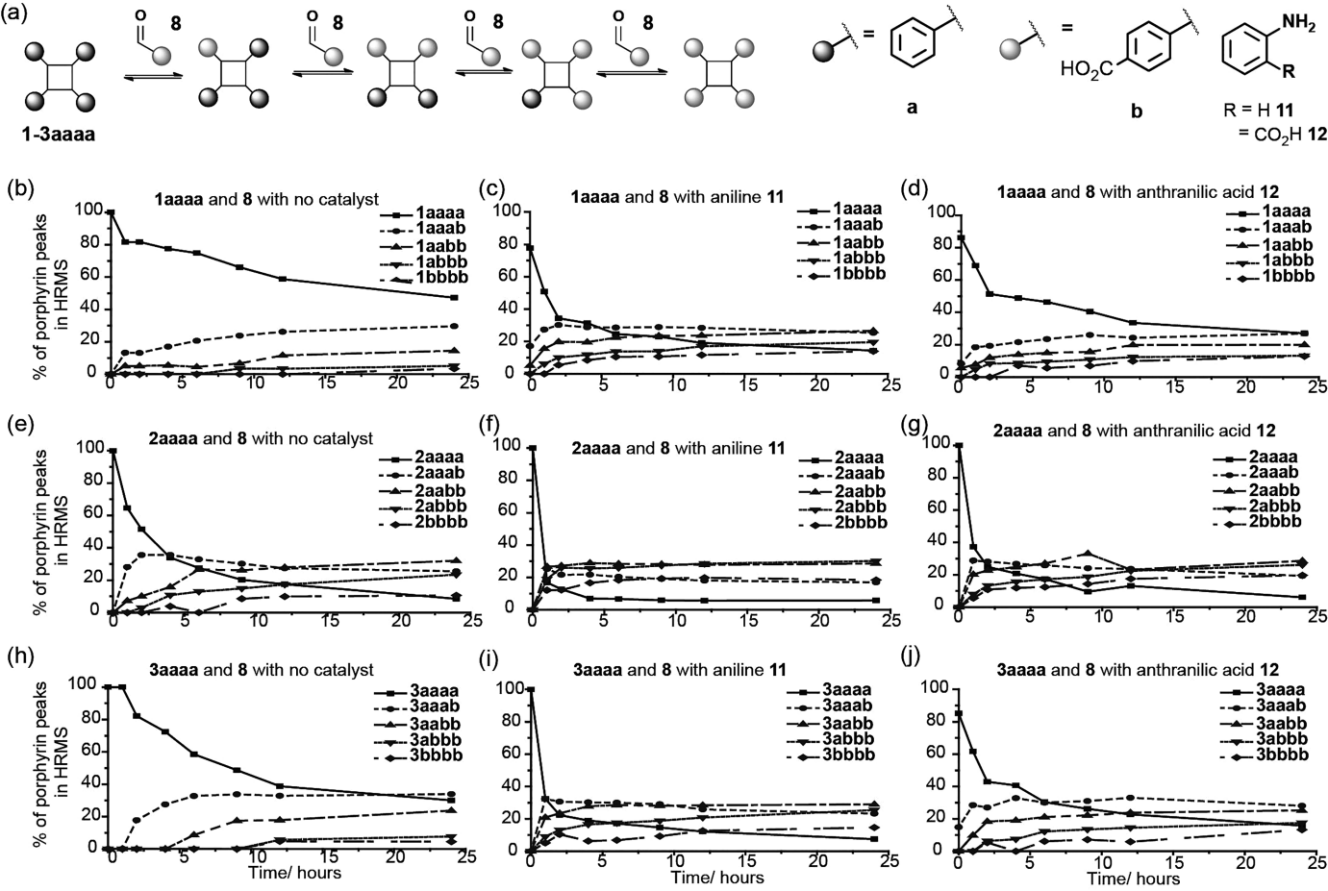
Effect of catalysis on porphyrin hydrazone exchange reactions; (a) schematic of reaction; (b), (c) and (d) serine hydrazone porphyrin **1aaaa**; (e), (f) and (g) aspartic acid hydrazone porphyrin **2aaaa**; and (h), (i) and (j) glutamic acid porphyrin **3aaaa**; (b), (e) and (h) no catalyst; (c), (f) and (i) aniline **11** (d), (g) and (j) anthranilic acid **12**, conditions: 100 µm hydrazone porphyrin **1**–**3aaaa**; 5 mm 4‐carboxybenzaldehyde **8**; no catalyst, 10 mm aniline **11** or 10 mm anthranilic acid **12**; (10 % DMSO in 5 mm NH_4_OAc, pH 6.75).

Nucleophilic catalysts have been used to promote hydrazone exchange reactions in biologically relevant media.^40^, ^42^, ^43^ Aniline **11** and anthranilic acid **12** were chosen, as they are cheap and relatively soluble. Hydrazone exchange reactions using **8** with the three porphyrin scaffolds **1**–**3aaaa** were performed with both catalysts; these led to an increase in the exchange rate in both cases with the former appearing to have a pronounced effect. Similar efforts to establish the effect of catalysts on the hydrazone exchange reaction using **9** and **10** again showed the catalysts increasing the rate of reaching equilibrium. A full comparison was not feasible given that **9** formed an insoluble imine on reaction with **11**, whereas **10** precipitated on reaction with **12**. We also considered catalyst loading. The effect of aniline concentration on exchange reactions with **10**, for hydrazone porphyrin scaffolds **1aaaa** and **2aaaa** was studied. Aldehyde **10** was chosen because it displayed the slowest equilibration rates in the absence of catalyst (Figure 2). From these data (see Supporting information) it was established that for <10 mm of **11**, hydrazone exchange was sluggish, but that significant rate increases could be achieved for ≥10 mm of **11**, hence this concentration was used in subsequent studies.

### Assembly of DCLs Based on Hydrazone‐Functionalized Porphyrins

Having established effective exchange of the benzaldehyde moiety for another aldehyde it was necessary to assess the extent to which a thermodynamic rather than kinetic distribution of products was obtained. The speciation of porphyrins reacted with 25 equivalents of aldehyde for 24 h followed by addition of a further 25 equivalents of the same aldehyde and reaction for 24 h was monitored. Using **10** and **11** as catalyst for all three hydrazone porphyrin scaffolds **1**–**3aaaa**, the product distribution was indeed changed at 48 h, (i.e. 24 h following addition of the second aliquot of aldehyde), with a shift to a greater number of hydrazone exchanges observed (see Supporting information).

**Figure 3:**
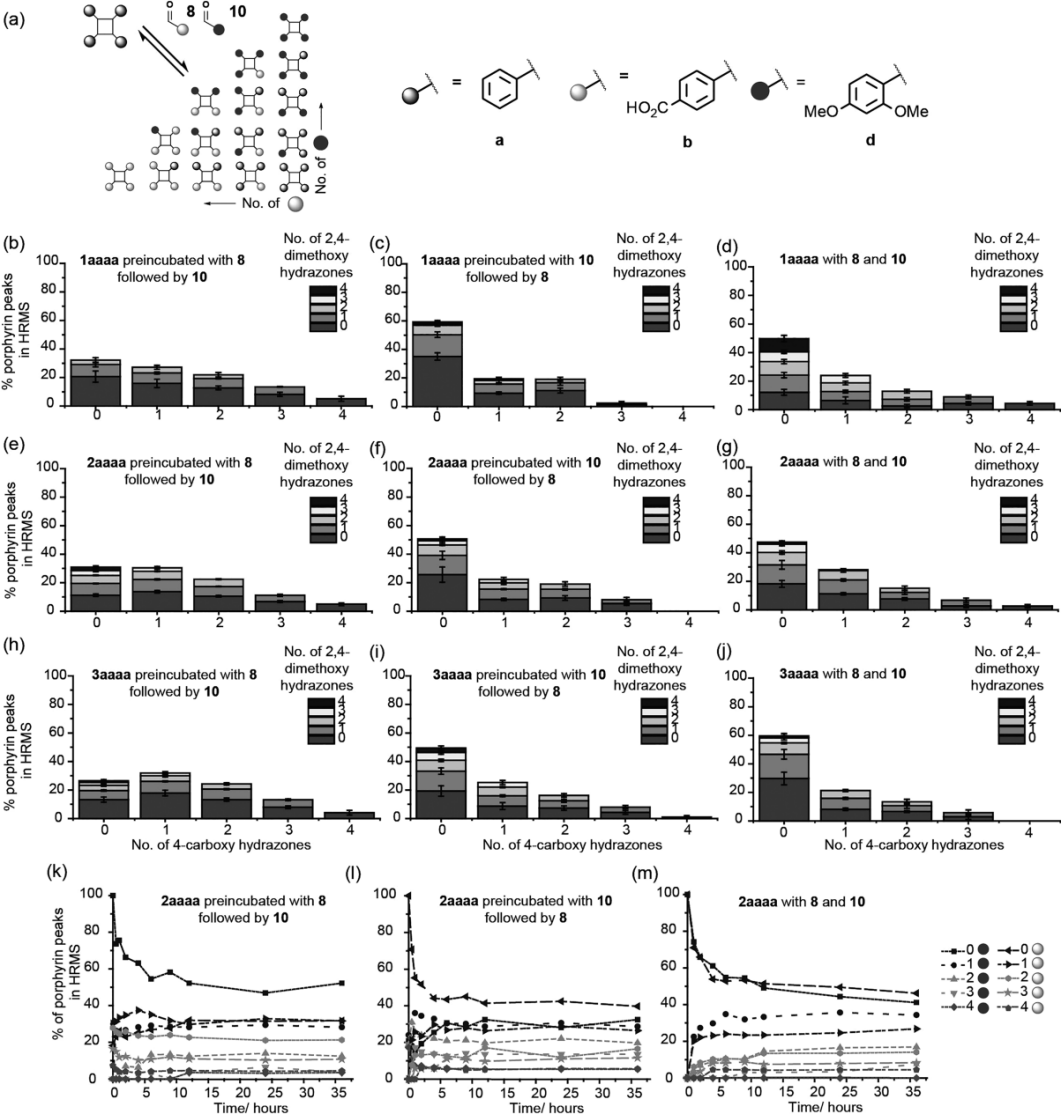
DCL generation; (a) schematic depicting process; (b–j) relative HRMS signal taken after 24 h incubation, pre‐incubated systems incubated with the first aldehyde for 24 h before addition of the second aldehyde (*n* = average of 5 separate incubations); (b–d) serine hydrazone porphyrin **1aaaa**; (e–g) aspartic acid hydrazone porphyrin **2aaaa**; (h–j) glutamic acid hydrazone porphyrin **3aaaa**; (b), (e) and (h) pre‐incubation with 4‐carboxy benzaldehyde **8**; (c), (f) and (i) pre‐incubation with 2,4‐dimethoxy benzaldehyde **10**; (d) (g), and (i) direct mixing of both aldehydes; (k–m) time for porphyrin hydrazone **2aaaa** exchanges to reach equilibrium with two aldehydes; (k) pre‐incubation with 4‐carboxy benzaldehyde **8**; (l) pre‐incubation with 2,4‐dimethoxy benzaldehyde **10**; (m) direct mixing of both aldehydes (conditions: 100 µm hydrazone porphyrin **1**–**3aaaa**, 2.5 mm each aldehyde, 10 mm aniline **11**, 10 % DMSO, 5 mm NH_4_OAc, pH 6.75).

Having established suitable conditions for thermodynamic control of product distribution, exchange reactions on the hydrazone‐functionalized porphyrins **1**–**3aaaa** were carried out using two aldehydes simultaneously to generate a DCL (Figure 3). Initially efforts focused on analyses of mixtures that had been preincubated with one aldehyde followed by addition of the other aldehyde with comparison to mixtures, in which the two aldehydes were present from the start: aniline **11** was used as a catalyst, and **8** as well as **10** were used with **1**–**3aaaa**. In all cases different hydrazone compositions were observed. With all three hydrazone porphyrin scaffolds **1**–**3aaaa**, pre‐incubation with **10** followed by addition of **8** gave a qualitatively similar distribution of products to that obtained by direct mixing of the two aldehydes, reinforcing the notion that a thermodynamic product distribution was obtained. There was, however, a more significant difference between the pre‐incubation with **8** and the direct mixing; some of the discrepancy may be attributed to limitations in the analyses, namely for mixtures with two aldehydes, discerning the signal for low abundance species in the mass spectrum becomes more challenging. However, it might also indicate the system did not reach equilibrium, or that non‐covalent associations in either of the two experiments generate a kinetic trap. Such behaviour has been observed in other dynamic combinatorial libraries^47^ and represents a key opportunity to explore systems chemistry.^48^ Nonetheless, the analyses described here clearly establish the proof‐of‐principle that porphyrin‐based hydrazones can be used to generate libraries of protein surface mimetics through hydazone exchange, and, that such libraries could in principle be used under the selection pressure of a protein to amplify high affinity protein‐surface binding ligands. To reinforce this observation, the timescale for equilibration in a DCL comprising a hydrazone‐functionalized porphyrin incubated with two aldehydes was assessed. Exemplary data for **2aaaa** are shown in Figure 3k–m. For both pre‐incubation (i.e. successive addition of aldehydes) and direct mixing (i.e. both aldehydes added simultaneously), equilibrium was reached within 12 hours consistent with the simpler experiments. Where both aldehydes were added together (Figure 3m) minor changes were still observed up to 35 h, however the rate of change was such that we considered further significant changes to library distribution would not be expected on a useful timescale.

## Conclusions

In summary, we have described a series of porphyrin‐functionalized scaffolds **1**–**3aaaa** and studied their hydrazone exchange reactions in aqueous buffer using simple aldehydes **8**–**10**. These studies have established effective conditions using an aniline catalyst, under which hydrazone exchange can be used to generate dynamic combinatorial libraries amenable to characterization by high resolution electrospray ionization mass spectrometry. Future studies will focus on elaborating the approach for the protein‐directed selection of high affinity protein‐surface mimetics using a dynamic combinatorial chemistry approach.

## Experimental Section

**General Considerations:** Reagents and solvents were purchased from major suppliers and used without further purification. Anhydrous chloroform, dichloromethane, and methanol were obtained from the in‐house solvent purification system, from Innovative Technology Inc. PureSolv®, other solvents used were of HPLC grade. Water for aqueous solutions was deionised.

Thin layer (silica) chromatography was performed using Merck Kiesegel 60 F_254_ 0.25 mm precoated aluminium plates. Product spots were visualised by colour and under UV light (254 nm and 365 nm). Flash column chromatography was performed using silica gel 60 (0.043–0.063 mm VWR or Sigma Aldrich) or alumina (Brockman I from Sigma Aldrich), unless otherwise stated silica gel was used and pressure was applied by means of head bellows.

^1^H NMR spectra were obtained on Bruker DPX 300 (300 MHz) Avance 500 (500 MHz) or DRX500 (500 MHz) spectrometers and referenced to either residual non‐deuterated solvent peaks or tetramethylsilane. ^13^C spectra were recorded on a Bruker DPX 300 (75 MHz) Bruker or an Avance 500 (126 MHz) and referenced to the solvent peak. ^1^H spectra are reported as follows: ^1^H NMR (spectrometer frequency, solvent): *δ* = ppm to 2 d.p. (multiplicity, *J* coupling constant in Hertz, number of protons, assignments). Chemical shifts are quoted in ppm with signal splitting recorded as singlet (s), doublet (d), triplet (t), quartet (q), multiplet (m), broad (br). Coupling constants, *J*, are measured to the nearest 0.1 Hz. Similarly, ^13^C spectra are reported as follows: *δ* (spectrometer frequency, solvent): *δ* ppm to one decimal place. Infrared spectra were recorded on a Perkin–Elmer Fourier‐Transfer spectrometer. Spectra were analysed neat and structurally important absorptions are quoted. Absorption maxima (ν̃_max_=) are quoted in wavenumbers (cm ^–1^). HPLC LC‐MS were recorded on a Bruker HCT ultra under electrospray ionisation (ESI) conditions. High resolution mass spectra were recorded on a Bruker Daltonics micrOTOF Premier Mass Spectrometer, under positive ESI conditions unless otherwise stated.

**4‐(7,12‐Bis{4‐[((1R)‐2‐(*tert*‐butoxy)‐1‐{*N′*‐[(*tert*‐butoxy)carbonyl]hydrazinecarbonyl}ethyl)carbamoyl]phenyl}‐17‐{4‐[((1S)‐2‐(*tert*‐butoxy)‐1‐{*N′*‐[(*tert*‐butoxy)carbonyl]hydrazinecarbonyl}ethyl)carbamoyl]phenyl}‐21,22,23,24‐tetraazapentacyclo[16.2.1.1^3^,^6^.1^8^,^11^.1^13^,^16^]tetracosa‐1,3(24),4,6,8,10,12,14,16(22),17,19‐undecaen‐2‐yl)‐*N*‐((1S)‐2‐(*tert*‐butoxy)‐1‐{*N′*‐[(*tert*‐butoxy)carbonyl]hydrazinecarbonyl}ethyl)benzamide (4):** Tetracarboxyphenylporphyrin (100 mg, 0.126 mmol), PyBOP (395 mg, 0.759 mmol), *tert*‐butyl 2‐(2‐amino‐3‐*tert*‐butoxypropanoyl)hydrazinecarboxylate, (209 mg, 0.759 mmol) and diisopropylethylamine (0.26 mL, 1.5 mmol) in anhydrous dimethylformamide (5 mL) were stirred under a nitrogen atmosphere for 18 h. The reaction mixture was then diluted with ethyl acetate (50 mL) and washed with saturated sodium hydrogen carbonate solution (50 mL), 1 m hydrochloric acid (50 mL) and brine (3 × 50 mL). The organic phase was dried (sodium sulphate) and concentrated to yield the crude product as a purple solid. This was purified by flash column chromatography (1:1 ethyl acetate/dichloromethane then ethyl acetate) to yield the product as a purple solid (142 mg, 0.078 mmol, 62 %). ^1^H NMR (500 MHz, CDCl_3_): *δ* = 1.29 (s, 36 H, *t*Bu), 1.45 (m, 36 H, *t*Bu), 3.51–3.63 (m, 8 H, β‐CH_2_), 4.07 (dt, *J* = 8.3, 4.7 Hz, 4 H, α‐CH), 4.83 (br. s, 4 H, NH), 6.40–6.57 (m, 4 H, NH), 8.15 (d, *J* = 7.5 Hz, 8 H, Ar‐H), 8.21 (d, *J* = 7.5 Hz, 8 H, Ar‐H), 8.40 (d, *J* = 8.3 Hz, 4 H, NH), 8.73 (br. s, 8 H, Pyr‐H) ppm. ^13^C NMR (101 MHz, MeOD): *δ* = 26.8, 27.5, 38.4, 53.3, 61.6, 74.0, 80.8, 106.6, 119.3, 125.8, 133.4, 134.4, 145.2, 156.2, 168.4, 170.8 ppm. IR (solid state): ν̃ = 3251 (N–H), 1696 (C=O carbamate), 1645 (C=O amide) cm^–1^. ESI‐MS *m/z* found 1820.9402 [M + 2H]^+^, [C_96_H_124_N_16_O_20_]^+^ calcd. 1820.9178.

**4‐(7,12‐Bis{4‐[((1R)‐2‐hydroxy‐1‐{*N′*‐[(1E)‐phenylmethylidene]hydrazinecarbonyl}ethyl)carbamoyl]phenyl}‐17‐{4‐[((1S)‐2‐hydroxy‐1‐{*N′*‐[(1E)‐phenylmethylidene]hydrazinecarbonyl}ethyl)carbamoyl]phenyl}‐21,22,23,24‐tetraazapentacyclo[16.2.1.1^3^,^6^.1^8^,^11^.1^13^,^16^]tetracosa‐1,3(24),4,6,8,10,12,14,16(22),17,19‐undecaen‐2‐yl)‐*N*‐((1S)‐2‐hydroxy‐1‐{*N′*‐[(1E)‐phenylmethylidene]hydrazinecarbonyl}ethyl)benzamide (1aaaa):** **4** (142 mg, 0.0780 mmol) in trifluoroacetic acid (4.5 mL), water (0.25 mL) and triisopropylsilane (0.25 mL) was stirred for 6 h. The solution was then concentrated to yield the deprotected hydrazide as a green solid (complete deprotection was confirmed by HRMS). The green solid was then redissolved in water (2 mL) and acetonitrile (2 mL) and benzaldehyde (2 drops) was added, the mixture was then stirred for 30 min. The precipitate was isolated and washed with water and acetonitrile to yield the product as a dark purple solid (30 mg, 0.019 mmol, 25 %). ^1^H NMR (500 MHz, [D_6_]DMSO): *δ* = 1.67 (br. s, 4 H, OH), 3.94 (br. s, 8 H, β‐CH_2_), 4.76 (br. s, 4 H, NH), 5.63 (br. s, 4 H, α‐CH), 7.51 (m, 8 H, Ar‐H), 7.63 (m, 8 H, Ar‐H), 7.75 (m, 4 H, Ar‐H), 7.95 (br. s, 4 H, Ar‐H), 8.12 (br. s, 4 H, Pyr‐H), 8.39 (br. s, 8 H, Ar‐H), 8.84 (m, 2 H, Ar‐H), 8.91 (br. s, 10 H, Ar‐H), 11.56 (br. s, 4 H, N=CH), 11.73 (br. s, 4 H, N=CH′) (*cis* and *trans* hydrazone isomers observed in 1:1 ratio). IR (solid state): ν̃ = 3212 (N–H), 1633 (C=O amide), 1608 (C=N) cm^–1^. ESI‐MS *m/z* found 1547.5750 [M + H]^+^, [C_88_H_75_N_16_O_12_]^+^: calcd. 1547.5340.

***tert*‐Butyl (3S)‐3‐{[4‐(7,12‐Bis{4‐[((1R)‐3‐(*tert*‐butoxy)‐1‐{*N′*‐[(*tert*‐butoxy)carbonyl]hydrazinecarbonyl}‐3‐oxopropyl)carbamoyl]phenyl}‐17‐{4‐[((1S)‐3‐(*tert*‐butoxy)‐1‐{*N′*‐[(*tert*‐butoxy)carbonyl]hydrazinecarbonyl}‐3‐oxopropyl)carbamoyl]phenyl}‐21,22,23,24‐tetraazapentacyclo[16.2.1.1^3^,^6^.1^8^,^11^.1^13^,^16^]tetracosa‐1,3(24),4,6,8,10,12,14,16(22),17,19‐undecaen‐2‐yl)phenyl]formamido}‐3‐{*N′*‐[(*tert*‐butoxy)carbonyl]hydrazinecarbonyl}propanoate (5):** Tetracarboxyphenylporphyrin (43 mg, 0.055 mmol), PyBOP (172 mg, 0.330 mmol), (*S*)‐*tert*‐butyl 2‐(2‐amino‐4‐*tert*‐butoxy‐4‐oxobutanoyl)hydrazinecarboxylate, (100 mg, 0.330 mmol) and anhydrous diisopropylethylamine (0.11 mL, 0.66 mmol) in anhydrous dimethylformamide (5 mL) were stirred under a nitrogen atmosphere for 18 h. The reaction mixture was then diluted with ethyl acetate (50 mL) and washed with saturated sodium hydrogen carbonate solution (50 mL), 1 m hydrochloric acid (50 mL) and brine (3 × 50 mL). The organic phase was dried (sodium sulphate) and concentrated to yield the crude product as a purple solid. This was purified by flash column chromatography (1:1 ethyl acetate/dichloromethane then ethyl acetate) to yield the product as a purple solid (103 mg, 0.0533 mmol, 97 %). ^1^H NMR (500 MHz, MeOD): *δ* = 1.53 (s, 36 H, *t*Bu), 1.55 (s, 36 H, *t*Bu), 2.95 (dd, *J* = 17.4, 8.0 Hz, 4 H, β‐C*H*H′), 3.10 (dd, *J* = 17.4, 4.8 Hz, 4 H, β‐CH*H*′), 5.30 (br. s, 4 H, α‐CH), 5.45 (d, *J* = 1.4 Hz, 4 H, NH), 8.21 (br. s, 8 H, Pyr‐H), 8.27 (d, *J* = 6.7 Hz, 8 H, Ar‐H), 8.73 (br. s, 8 H, NH), 9.02 (d, *J* = 6.7 Hz, 8 H, Ar‐H). ^13^C NMR (101 MHz, MeOD + 10 % CDCl_3_): *δ* = 27.4, 27.6, 37.2, 49.3, 80.9, 81.5, 119.3, 125.9, 133.2, 134.4, 145.4, 149.5, 156.3, 168.4, 170.2, 171.3, 185.1 ppm. IR (solid state): ν̃ = 3275 (N–H), 1723 (C=O ester), 1711 (C=O carbamate), 1647 (C=O amide) cm^–1^. ESI‐MS *m/z* found 1931.9891 [M + H]^+^, [C_100_H_123_N_16_O_24_]^+^: calcd. 1931.8896.

**3(S)‐3‐{[4‐(7,12‐Bis{4‐[((1R)‐2‐carboxy‐1‐{*N′*‐[(1E)‐phenylmethylidene]hydrazinecarbonyl}ethyl)carbamoyl]phenyl}‐17‐{4‐[((1S)‐2‐carboxy‐1‐{*N′*‐[(1E)‐phenylmethylidene]hydrazinecarbonyl}ethyl)carbamoyl]phenyl}‐21,22,23,24‐tetraazapentacyclo[16.2.1.1^3^,^6^.1^8^,^11^.1^13^,^16^]tetracosa‐1,3(24),4,6,8,10,12,14,16(22),17,19‐undecaen‐2‐yl)phenyl]formamido}–3‐{*N′*‐[(1E)‐phenylmethylidene]hydrazinecarbonyl}propanoic Acid (2aaaa):** **5** (50 mg, 0.026 mmol) in trifluoroacetic acid (4.5 mL), water (0.25 mL) and triisopropylsilane (0.25 mL) was stirred for 6 h. The solution was then concentrated to yield deprotected hydrazide as a green solid (complete deprotection was confirmed by HRMS). The green solid was then redissolved in water (2 mL) and acetonitrile (2 mL) and benzaldehyde (2 drops) added, the mixture was then stirred for 30 min. The precipitate was isolated and washed with water and acetonitrile to yield the product as a dark purple solid (16 mg, 0.0096 mmol, 37 %). ^1^H NMR (500 MHz, [D_6_]DMSO): *δ* = –2.98 to –2.85 (br. s, 2 H, NH), 2.78–3.06 (m, 8 H, β‐CH_2_), 5.06 (m, 4 H, α‐CH), 5.87 (m, 4 H, NH), 7.46 (d, *J* = 6.7 Hz, 8 H, Ar‐H), 7.73 (d, *J* = 6.8 Hz, 4 H, Ar‐H), 7.76 (d, *J* = 6.8 Hz, 4 H, Ar‐H), 8.06 (m, 4 H, NH), 8.34 (m, 16 H, Ar‐H, Pyr‐H), 8.85 (br. s, 8 H, Ar‐H), 9.08 (m, 2 H, Ar‐H), 9.14–9.23 (m, 2 H, Ar‐H), 11.54 (br. s, 2 H, N=CH), 11.67 (br. s, 2 H, N=CH′), 12.46 (br. s, 4 H, OH) (*cis* and *trans* isomers of hydrazone present in 1:1 ratio). IR (solid state): ν̃ = 3270 (N–H), 1714 (C=O acid), 1643 (C=O amide), 1607 (C=N) cm^–1^. ESI‐MS *m/z* found 1659.5058 [M + H]^+^, [C_92_H_75_N_16_O_16_]^+^: calcd. 1659.5547.

***tert*‐Butyl (4S)‐4‐{[4‐(7,12‐**bis** {4‐[((1R)‐4‐(*tert*‐butoxy)‐1‐{*N′*‐[(*tert*‐butoxy)carbonyl]hydrazinecarbonyl}‐4‐oxobutyl)carbamoyl]phenyl}‐17‐{4‐[((1S)‐4‐(*tert*‐butoxy)‐1‐{*N′*‐[(*tert*‐butoxy)carbonyl]hydrazinecarbonyl}‐4‐oxobutyl)carbamoyl]phenyl}‐21,22,23,24‐tetraazapentacyclo[16.2.1.1^3^,^6^.1^8^,^11^.1^13^,^16^]tetracosa‐1,3(24),4,6,8,10,12,14,16(22),17,19‐undecaen‐2‐yl)phenyl]formamido}‐4‐{*N′*‐[(*tert*‐butoxy)carbonyl]hydrazinecarbonyl}butanoate (6):** Tetracarboxyphenylporphyrin (42 mg, 0.053 mmol), PyBOP (164 mg, 0.315 mmol), (*S*)‐*tert*‐butyl 2‐(2‐amino‐5‐*tert*‐butoxy‐5‐oxopentanoyl)hydrazinecarboxylate, (100 mg, 0.315 mmol) and diisopropylethylamine (0.11 mL, 0.63 mmol) in anhydrous dimethylformamide (5 mL) were stirred under nitrogen for 18 h. The reaction mixture was then diluted with ethyl acetate (50 mL) and washed with saturated sodium hydrogen carbonate solution (50 mL), 1 m hydrochloric acid (50 mL) and brine (3 × 50 mL). The organic phase was dried (sodium sulphate) and concentrated to yield the crude product as a purple solid. This was purified by flash column chromatography (1:1 ethyl acetate/dichloromethane then ethyl acetate) to yield the product as a purple solid (65 mg, 0.033 mmol, 62 %). ^1^H NMR (500 MHz, MeOD): *δ* = 1.51 (s, 36 H, *t*Bu), 1.54 (s, 36 H, *t*Bu), 2.34 (m, 8 H, β‐CH_2_), 2.63 (m, 8 H, γ‐CH_2_), 3.35 (br. s, 4 H, NH), 3.39 (s, 4 H, NH), 4.92 (br. s, 4 H, α‐CH), 8.00 (br. s, 8 H, Ar‐H), 8.18 (br. s, 8 H, Ar‐H), 8.61 (m, 8 H, Pyr‐H). ^13^C NMR (101 MHz, MeOD + 10 % CDCl_3_): *δ* = 27.1, 27.5, 27.7, 31.7, 51.9, 81.0, 119.4, 125.9, 133.2, 134.5, 145.4, 149.5, 156.2, 167.5, 168.4, 170.3, 200.0 ppm. IR (solid state): ν̃ = 3275 (N–H), 1723 (C=O ester), 1643 (C=O carbamate), 1608 (C=O amide) cm^–1^. ESI‐MS *m/z* found 1988.0341 [M + H]^+^, [C_104_H_131_N_16_O_24_]^+^: calcd. 1987.9522.

**(4S)‐4‐{4‐[7,12‐Bis(4‐[((1R)‐3‐carboxy‐1‐{*N′*‐[(1E)‐phenylmethylidenehydrazinecarbonyl]propyl}carbamoyl)phenyl]‐17‐{4‐[((1S)‐3‐carboxy‐1‐{*N′*‐[(1E)‐phenylmethylidene]hydrazinecarbonyl}propyl)carbamoyl]phenyl}‐21,22,23,24‐tetraazapentacyclo[16.2.1.1^3^,^6^.1^8^,^11^.1^13^,^16^]tetracosa‐1,3(24),4,6,8,10,12,14,16(22),17,19‐undecaen‐2‐yl)phenyl]formamido}‐4‐{*N′*‐[(1E)‐phenylmethylidene]hydrazinecarbonyl}butanoic Acid (3aaaa):** **6** (30 mg, 0.015 mmol) in trifluoroacetic acid (4.5 mL), water (0.25 mL) and triisopropylsilane (0.25 mL) was stirred for 6 h. The solution was then concentrated to yield the deprotected hydrazide porphyrin as a green solid (complete deprotection was confirmed by HRMS). The green solid was then redissolved in water (2 mL) and acetonitrile (2 mL) and benzaldehyde (2 drops) was added, the mixture was then stirred for 30 min. The precipitate was isolated and washed with water and acetonitrile to yield the product as a dark purple solid (25 mg, 0.0093 mmol, 62 %). ^1^H NMR (500 MHz, [D_6_]DMSO): *δ* = –2.94 (br. s, 2 H, NH), 0.79 (m, 4 H, β‐C*H*H′), 1.36 (m, 4 H, β‐CH*H*′), 2.07 (m, 8 H, γ‐CH_2_), 4.59 (br. s, 4 H, α‐CH), 5.48 (br. s, 4 H, NH), 7.45 (br. s, 8 H, Ar‐H), 7.56–7.67 (m, 4 H, Ar‐H), 7.73 (m, 4 H, Ar‐H), 7.82 (m, 4 H, Ar‐H), 7.87–7.98 (m, 4 H, Ar‐H), 8.09 (br. s, 4 H, NH), 8.36 (br. s, 8 H, Pyr‐H), 8.83 (br. s, 8 H, Ar‐H), 8.94 (m, 2 H, Ar‐H), 9.04 (br. s, 2 H, Ar‐H), 11.52 (m, 2 H, N=CH), 11.68 (m, 2 H, N=CH′), 12.33 (br. s, 4 H, OH) (*cis* and *trans* isomers of hydrazone observed in 1:1 ratio). IR (solid state): ν̃ = 3283 (N–H), 1633 (C=O acid), 1607 (C=O amide), 1529 (C=N) cm^–1^. ESI‐MS: *m/z* = found 1715.5801 [M + H]^+^, [C_96_H_83_N_16_O_16_]^+^: calcd. 1715.6173.

**Hydrazone Exchange Studies:** Hydrazone exchange reactions were carried out in HPLC vials and were followed using high resolution mass spectrometry on a Bruker Daltonics micrOTOF Premier Mass Spectrometer, using 10 µL injections and summing the masses over a range of 1.0 to 3.0 min. The intensity of the maximum peak for each of the successive hydrazone exchanges was taken and the percentage associated with each of the porphyrin peaks calculated.

Hydrazone functionalised porphyrins **1**–**3aaaa** were made up to 5 mm concentration in DMSO and were stored in plastic Eppendorf tubes. 1 m stocks of catalyst (**11** and **12**) and aldehydes (**8**, **9**, and **10**) were made up in DMSO.

In all exchange reactions the hydrazone functionalised porphyrin **1**–**3aaaa** in DMSO was added to a solution of the aldehyde and catalyst to give a total concentration of 10 % DMSO in 5 mm ammonium acetate buffer, pH 6.75, to a final porphyrin concentration of 100 µm and stated concentrations of other components. For time courses, mass spectra were obtained at appropriates time points (usually 0.5, 1, 2, 4, 6, 9, 12 and 24 h). For measurements at single time points, mass spectra were obtained after 24 h incubation. For pre‐incubated samples, the pre‐incubated mixture was left for 24 h, and a mass spectrum obtained, prior to addition of further components.

## Supporting information

Supporting Information
