# Supplementary material for: Generation of Dynamic Combinatorial Libraries Using Hydrazone‐Functionalized Surface Mimetics
Source: European J Org Chem. 2018 Apr 25;2018(16):1872–9. doi: 10.1002/ejoc.201800022 (PMC5947633; doi:10.1002/ejoc.201800022)
Supplement: Supplementary file 1 — Supporting Information [file EJOC-2018-1872-s001.pdf]

*Eur. J. Org. Chem.* ISSN 1099–0690

https://doi.org/10.1002/ejoc.201800022

## ***SUPPORTING INFORMATION***

***Title:*** Generation of Dynamic Combinatorial Libraries Using Hydrazone-Functionalized Surface Mimetics
***Author(s):*** Sarah H. Hewitt and Andrew J. Wilson*

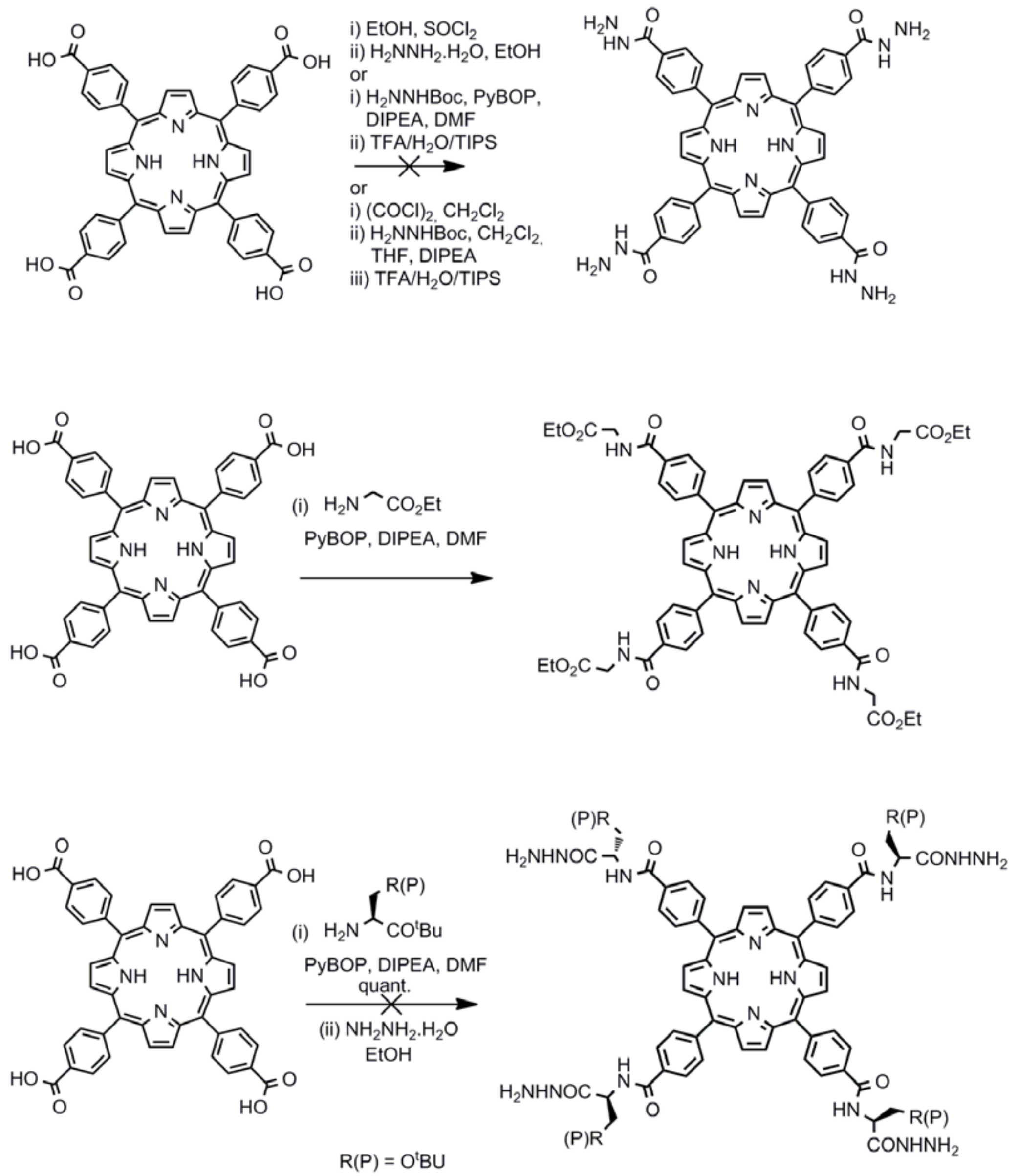


**Scheme ESI 1.** Alternative unsuccessful syntheses of hydrazide functionalized porphyrins studied in this work

## ***Tert*-butyl 2- (2- (((9H-fluoren-9-yl) methoxy) carbonylamino) -3- *tert*-butoxypropanoyl) hydrazine carboxylate**

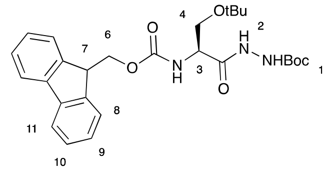


Fmoc-Ser (O$^t$Bu) COOH (5.00 g, 13.0 mmol), *tert*-butyl carbazate (5.17 g, 39.1 mmol), HATU (5.44 g, 14.3 mmol) and di*iso*propylethylamine (9.02 mL, 52.0 mmol) were stirred in anhydrous

dimethylformamide (20 mL) under a nitrogen atmosphere for 18 hours. The solution was then diluted with ethyl acetate (300 mL) and washed with saturated sodium hydrogen carbonate solution (200 mL), 1 M hydrochloric acid (200 mL) and brine (3 × 500 mL) to yield the crude product as an off-white solid. This was purified by flash column chromatography (20 % ethyl acetate in dichloromethane) to yield the product as a white solid (4.32 g, 8.68 mmol, 67 %); $^{1}H$ NMR (500 MHz, $CDCl_3$) δ ppm 1.24 (s, 9 H, H1/H5), 1.50 (s, 9 H, H1/H5), 3.48 (m, 1 H, H2+H3), 3.74 (q, $J$ = 6.5 Hz, 2 H, H4), 4.25 (t, $J$ = 7.0 Hz, 1 H, H7), 4.44 (d, $J$ = 7.0 Hz, 2 H, H6), 5.70 (br. s, 1 H, NH), 6.50 (br. s, 1 H, NH), 7.34 (t, $J$ = 7.6 Hz, 2 H, Fmoc), 7.43 (t, $J$ = 7.6 Hz, 2 H, Fmoc), 7.62 (dd, $J$ = 7.6, 3.0 Hz, 2 H, Fmoc), 7.79 (d, $J$ = 7.6 Hz, 2 H, Fmoc), 8.42 (br. s, 1 H, NH); $^{13}C$ NMR (75 MHz, DMSO-$d_6$) δ ppm 27.2, 28.0, 46.6, 54.2, 61.4, 61.8, 65.8, 72.9, 120.0, 127.0, 127.6, 125.4, 140.7, 143.7, 143.8, 155.0, 155.8; IR (solid state, $cm^{-1}$) 3297 (N-H), 3256 (N-H), 1714 (C=O carbamate), 1688 (C=O amide); ESI-MS $m/z$ found 520.2422 $[M+Na]^+$, $[C_{27}H_{35}N_3O_6Na]^+$ requires 520.2424

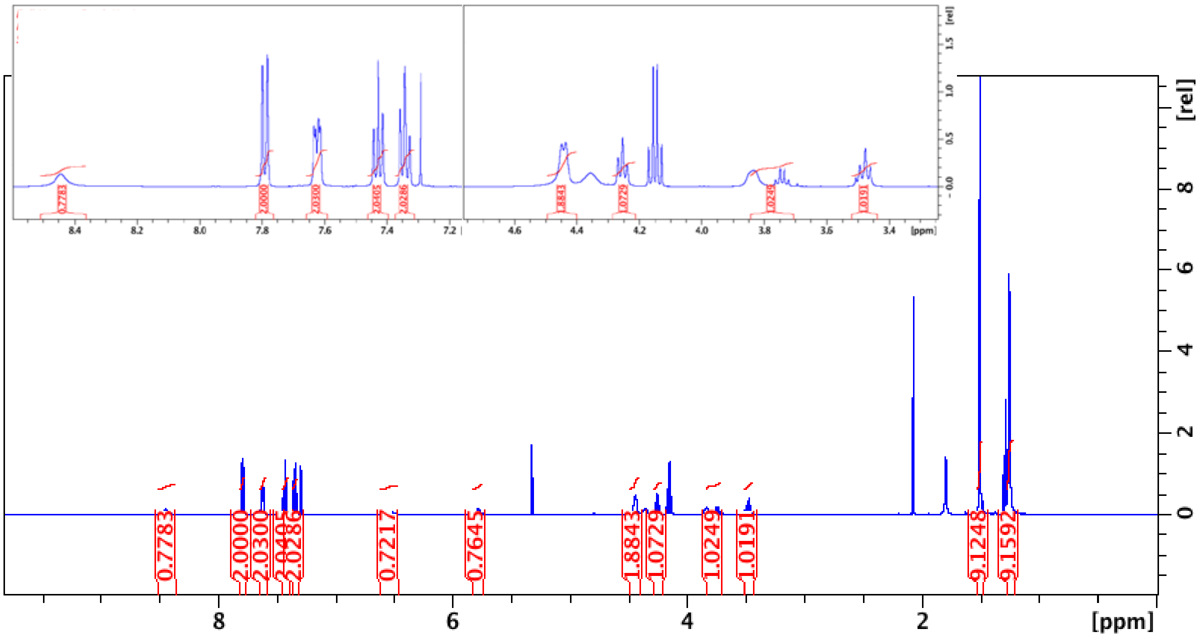

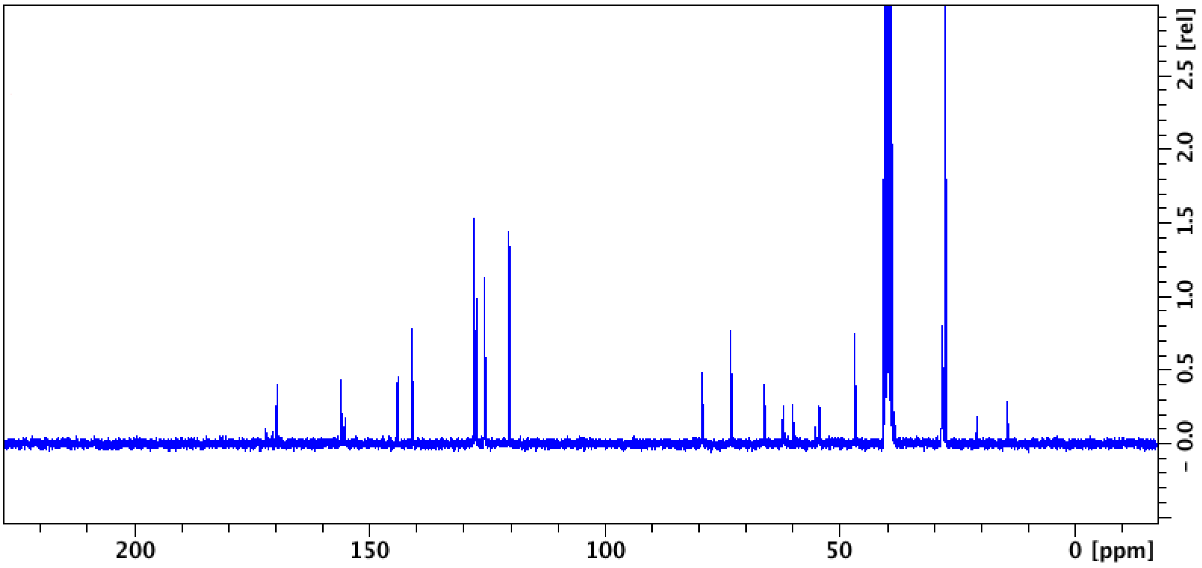


**Tert-butyl 2-(2-amino-3-*tert*-butoxypropanoyl)hydrazinecarboxylate**

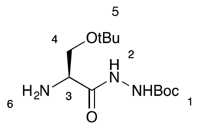


*Tert*-butyl 2-(2-(((9H-fluoren-9-yl)methoxy)carbonylamino)-3-*tert*-butoxypropanoyl) hydrazine carboxylate (1.00 g, 2.00 mmol) in 20 % diethylamine in acetonitrile was stirred for 16 hours. The reaction mixture was concentrated, and redissolved in a minimal amount of ethyl acetate and precipitated with hexane. The suspension was filtered through a celite pad and washed with hexane. The celite pad was then washed with dichloromethane and methanol, and this filtrate concentrated to yield the product as an off-white waxy solid (490 mg, 1.78 mmol, 88 %); $^{1}$H NMR (500 MHz, $CDCl_3$) δ ppm 1.18 - 1.28 (m, 9 H, H1/H5), 1.50 (s, 9 H, H1/H5), 1.66 - 1.99 (m, 4 H, H6+H2+ N<u>H</u>Boc), 3.55 (dd, $J$ = 4.7, 3.1 Hz, 1 H, H3), 3.59 - 3.68 (m, 2 H, H4/H4'); $^{13}$C NMR (101 MHz, MeOD) δ ppm 26.3, 27.2, 42.1, 63.2, 73.4, 80.6, 156.3. 172.5; IR (solid state, cm$^{-1}$) 3368 (N-H), 3242 (N-H), 1720 (C=O carbamate), 1692 (C=O amide); ESI-MS *m/z* found 276.1923 $[M+H]^+$, $[C_{12}H_{26}N_3O_4]^+$ requires 276.1923

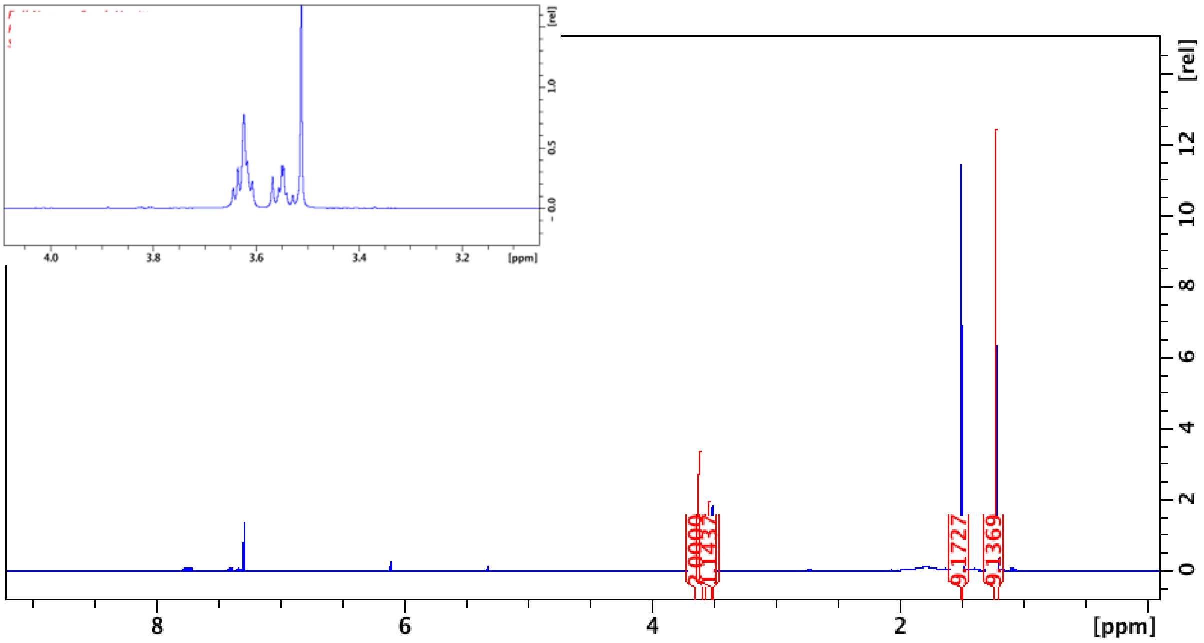


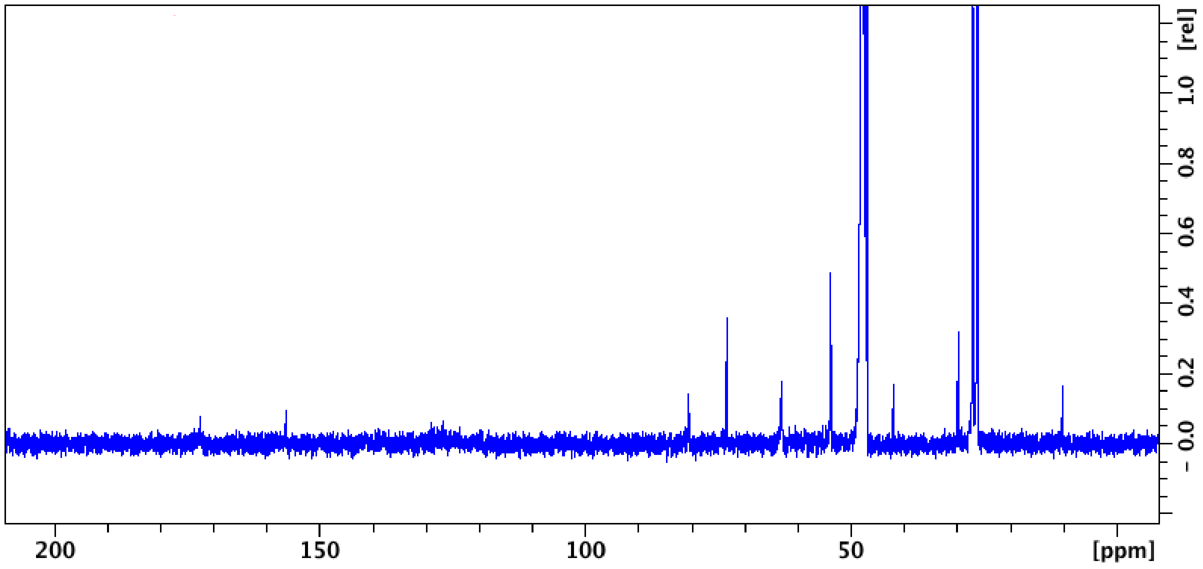


**(S)-*tert*-butyl 2-(2-(((9H-fluoren-9-yl) methoxy) carbonylamino)-4-*tert*-butoxy-4-oxobutanoyl)hydrazinecarboxylate**

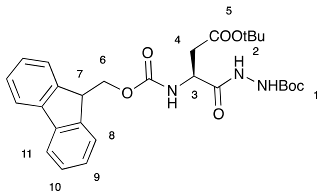


Fmoc- Asp(O$^t$Bu)COOH (1.50 g, 3.65 mmol), *tert*-butyl carbazate (1.45 g, 10.9 mmol), HATU (1.53 g, 4.00 mmol) and di*iso*propylethylamine (1.27 mL, 7.30 mmol) in anhydrous dimethylformamide (10 mL) were stirred for 16 hours under a nitrogen atmosphere. The solution was then diluted with ethyl acetate (100 mL) and washed with saturated sodium hydrogen carbonate solution (100 mL), 1 M hydrochloric acid (100 mL), brine (3 × 200 mL)

and ammonium hydroxide solution (100 mL). The organic phase was dried (sodium sulfate) and concentrated to yield the product as a white solid (2.02 g, 3.85 mmol, quant.); $^{1}H$ NMR (500 MHz, $CDCl_3$) δ ppm 1.48 (s, 9 H, H1/H5), 1.49 (s, 9 H, H1/H5), 2.69 (m, 1 H, H4), 2.91 (m, 1 H, H4'), 4.25 (t, *J* = 7.5 Hz, 1 H, H7), 4.41 - 4.54 (m, 1 H, H3) 4.57 - 4.68 (m, 1 H, NH) 5.98 (d, *J* = 7.5 Hz, 1 H, H6), 6.40 (br. s, 1 H, NH), 7.34 (t, *J* = 7.5 Hz, 2 H, Fmoc) 7.43 (t, *J* = 7.5 Hz, 2 H, Fmoc), 7.61 (dd, *J* = 7.5, 3.7 Hz, 2 H, Fmoc), 7.79 (d, *J* = 7.5 Hz, 2 H, Fmoc), 8.22 (br. s, 1 H, NH); $^{13}C$ NMR (75 MHz, DMSO-$d_6$) δ ppm 27.4, 27.7, 28.0, 31.3, 46.6, 52.3, 65.7, 79.7, 120.1, 125.3, 127.1, 127.6, 140.7, 143.8, 155.1, 155.8, 171.0, 171.6; IR (solid state, $cm^{-1}$) 3285 (N-H), 1695 (C=O amide); ESI-MS *m/z* found 548.2265 $[M+Na]^+$, $[C_{28}H_{35}N_3O_7Na]^+$ requires 548.2373

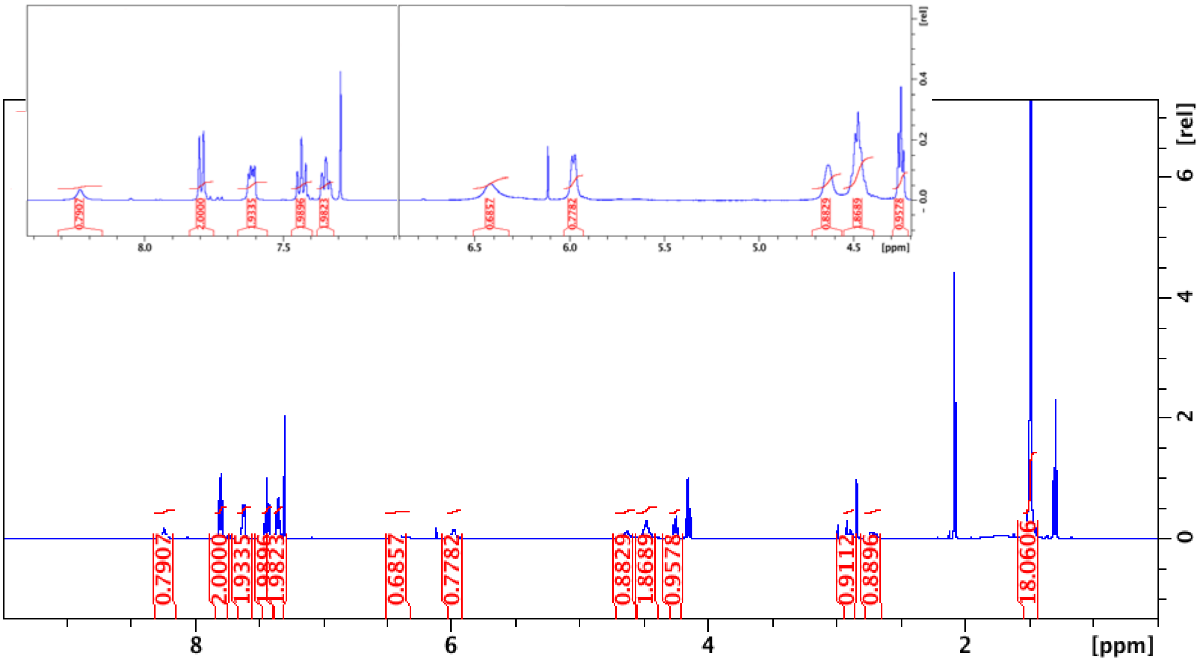


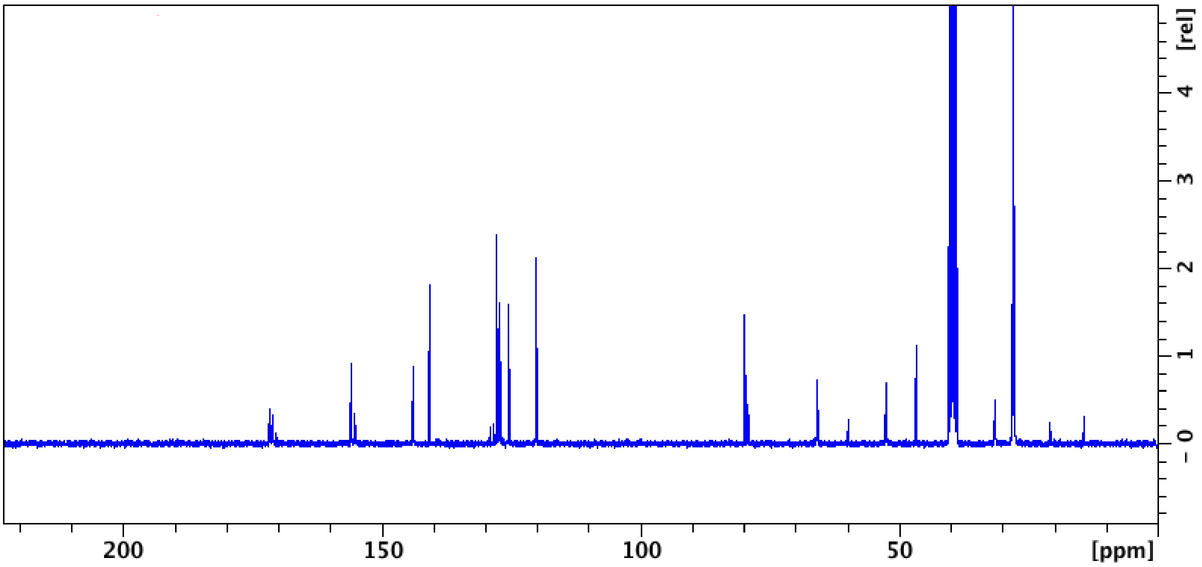

**(S)-*tert*-butyl 2-(2-amino-4-*tert*-butoxy-4-oxobutanoyl) hydrazinecarboxylate**

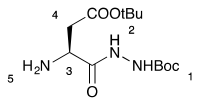


(S)-*tert*- butyl 2- (2- (((9H-fluoren-9-yl) methoxy) carbonyl amino)-4 -*tert*- butoxy- 4-oxobutanoyl) hydrazine carboxylate (1.85 g, 3.52 mmol) in 20 % diethylamine in acetonitrile (50 mL) was stirred for 16 hours. The reaction solution was then concentrated and the resulting residue redissolved in a minimal amount of ethyl acetate, and precipitated by addition of hexane. The slurry was then filtered through celite. The celite pad was then washed with dichloromethane and methanol and this filtrate concentrated to yield the product as an off-white waxy solid (1.03 g, 3.40 mmol, 96%); $^{1}$H NMR (500 MHz, $CDCl_3$) δ ppm 1.48 (s, 9 H, H1/H2), 1.50 (s, 9 H, H1/H2), 2.59 (dd, *J* = 16.7, 8.1 Hz, 1 H, H4), 2.82 (dd, *J* = 16.7, 3.7 Hz, 1 H, H4'), 3.16 (br. s, 1 H, H3), 3.91 (br. s, 2 H, H6), 6.38 (br. s, 1 H, H2); $^{13}$C NMR (101 MHz, MeOD) δ ppm 23.5, 25.8, 27.0, 36.7, 76.7, 81.1, 155.4, 170.4, 172.3; IR (solid state, cm$^{-1}$) 3274 (N-H), 1707 (C=O ester); ESI-MS *m/z* found 326.1685 $[M+Na]^+$, $[C_{13}H_{25}N_3O_5Na]^+$ requires 326.1686

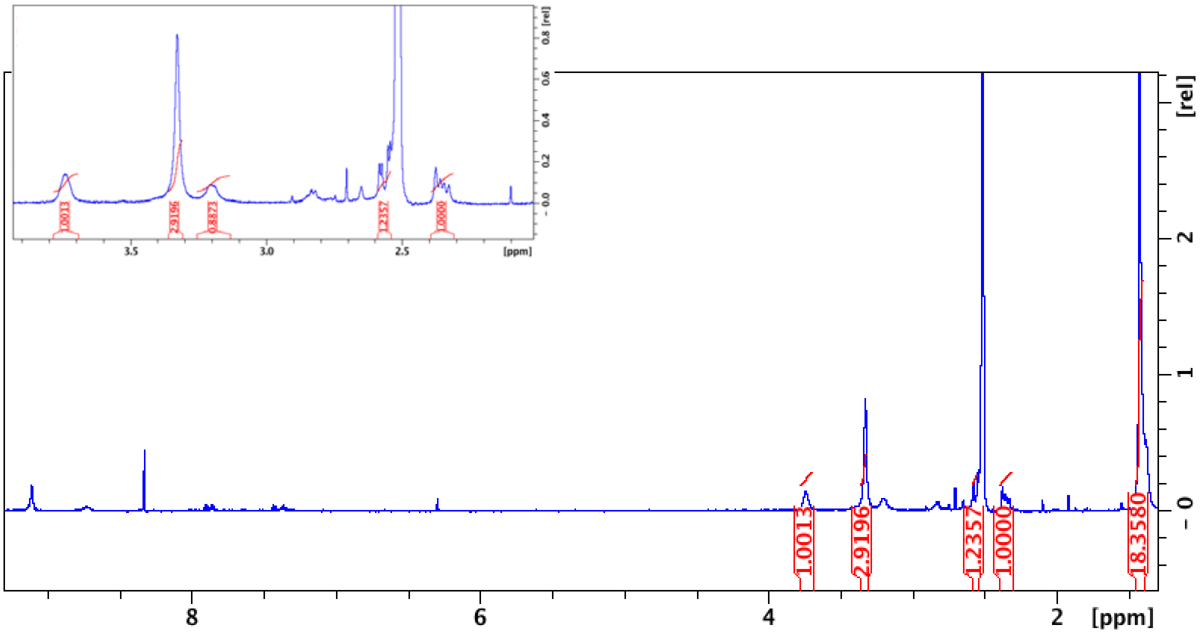

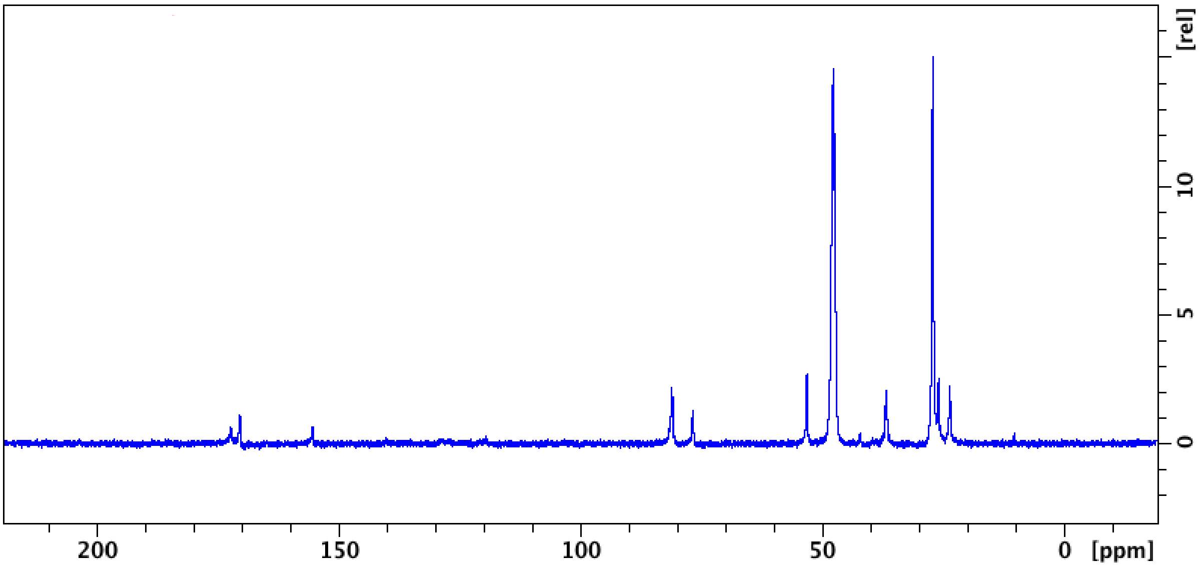


**(S)-*tert*-butyl 2-(2- (((9H-fluoren-9-yl) methoxy) carbonylamino)-5- *tert*- butoxy-5-oxopentanoyl) hydrazinecarboxylate**

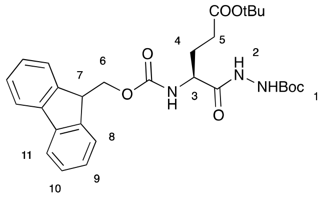


Fmoc Glu(O$^t$Bu)COOH (3.00 g, 7.05 mmol), HATU (2.95 g, 7.76 mmol), *tert*-butyl carbazate (2.80 g, 21.2 mmol) and di*iso*propylethylamine (2.46 mL, 14.1 mmol) in anhydrous dimethylformamide (20 mL) were stirred under a nitrogen atmosphere for 18 hours. The solution was then diluted with ethyl acetate (100 mL), and washed with saturated sodium hydrogen carbonate solution (100 mL), 1 M hydrochloric acid (100 mL), brine (3 × 200 mL) and ammonium hydroxide solution (100 mL). The organic phase was dried (sodium sulfate) and concentrated to yield the product as a white solid (3.85 g, 7.13 mmol, 92 %); $^1$H NMR (500 MHz, $CDCl_3$) δ ppm 1.47 (s, 9 H, H1/$^t$Bu), 1.48 (s, 9 H, H1/$^t$Bu), 2.01 (dq, *J* =14.0, 8.0 Hz, 1 H, H4), 2.15 (m, 1 H, H4'), 2.46 (m, 2 H, H5), 4.22 (t, *J* = 6.8 Hz, 1 H, H7), 4.32 (m, 1 H, H3), 4.40 (d, *J* = 6.8 Hz, 2 H, H6), 5.92 (br. s, 1 H, NH), 6.63 (br. s, 1 H, NH), 7.32 (t, *J* = 7.2 Hz, 2 H, Fmoc), 7.41 (t, *J* = 7.2 Hz, 2 H, Fmoc), 7.61 (dd, *J* = 7.2, 4.0 Hz, 2 H, Fmoc), 7.78 (d, *J* = 7.2 Hz, 2 H, Fmoc), 8.45 (m, 1 H, NH); $^{13}$C NMR (75 MHz, DMSO-$d_6$) δ ppm 27.7, 28.0, 31.3, 46.6, 52.3, 65.7, 79.1, 79.7, 120.0, 125.3, 127.0, 127.6, 140.7, 143.7, 143.9, 155.1, 155.8, 170.3, 171.0; IR (solid state, cm$^{-1}$) 3276 (N-H), 1692 (C=O amide); ESI-MS *m/z* found 562.2529 $[M+Na]^+$, $[C_{29}H_{37}N_3O_7Na]^+$ requires 562.2529

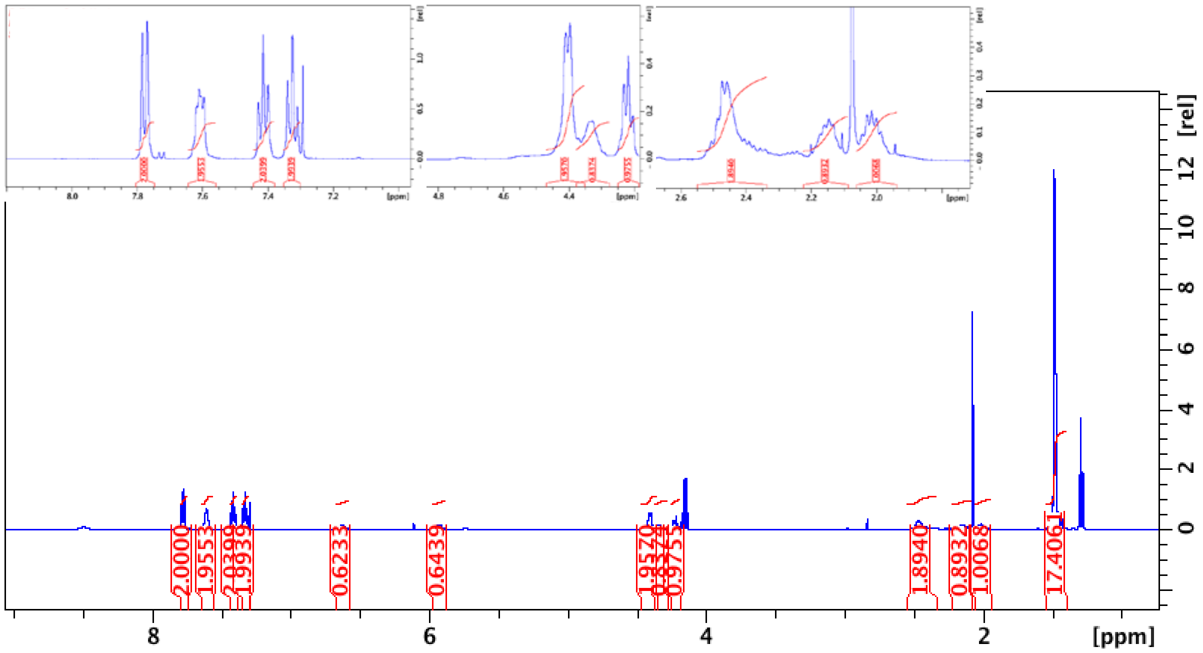

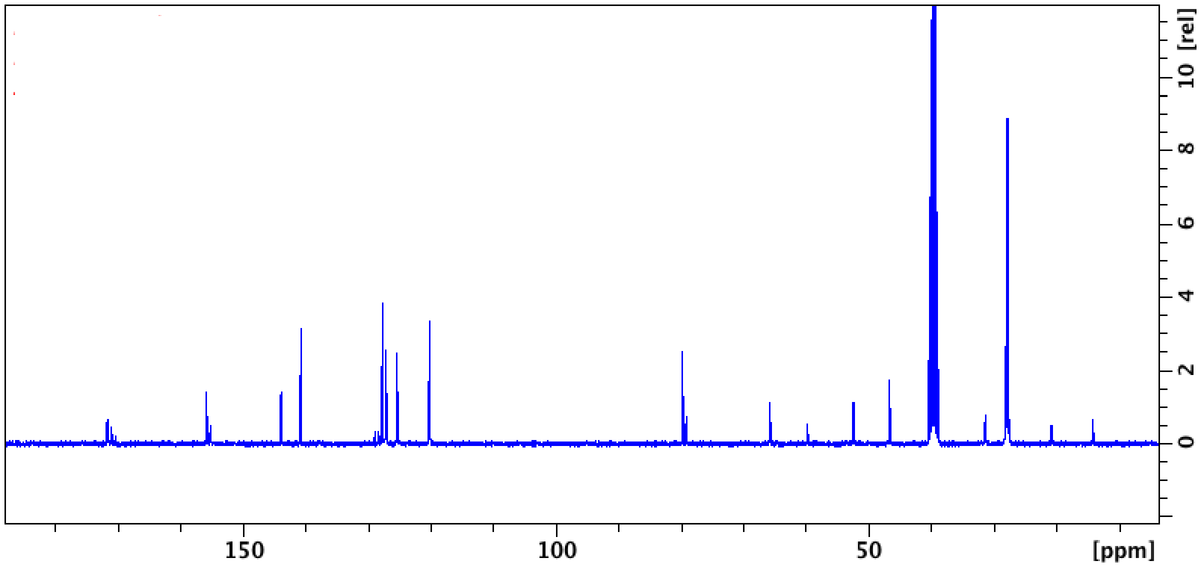

**(S)-*tert*-butyl 2-(2-amino-5-*tert*-butoxy-5-oxopentanoyl)hydrazinecarboxylate**

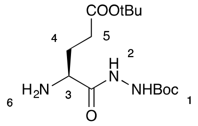


(S)-*tert*-butyl 2-(2-(((9H-fluoren-9-yl) methoxy) carbonylamino) -5- *tert*- butoxy-5-oxopentanoyl) hydrazinecarboxylate (650 mg, 1.20 mmol) in 20 % diethylamine in acetonitrile (50 mL) was stirred for 16 hours. The reaction solution was then concentrated and the resulting residue redissolved in a minimal amount of ethyl acetate, and precipitated by addition of hexane. The slurry was then filtered through celite. The celite pad was then washed with dichloromethane and methanol and this filtrate concentrated to yield the product as an off-white waxy solid (257 mg, 0.811 mmol, 68 %); $^{1}H$ NMR (500 MHz, $CDCl_3$) δ ppm 1.45 (s, 9 H,

H1/tBu), 1.48 (s, 9 H, H1/tBu), 1.85 (dq, *J* = 14.0, 7.0 Hz, 1 H, H4), 2.10 (dq, *J* = 14.0, 7.0 Hz, 1 H, H4'), 2.41 (app. t, *J* = 7.0 Hz, 2 H, H5), 3.51 (app. t, *J* = 7.0 Hz, 1 H, H3); $^{13}$C NMR (75 MHz, DMSO-d$_6$) δ ppm 27.7, 28.0, 30.6, 31.3, 48.5, 52.7, 79.4, 155.2, 172.2, 174.4; IR (solid state, cm$^{-1}$) 3293 (N-H), 1716 (C=O ester), 1595 (C=O amide); ESI-MS *m/z* found 318.2036 [M+H]$^+$, [$C_{14}H_{27}N_3O_5$]$^+$ requires 318.2029

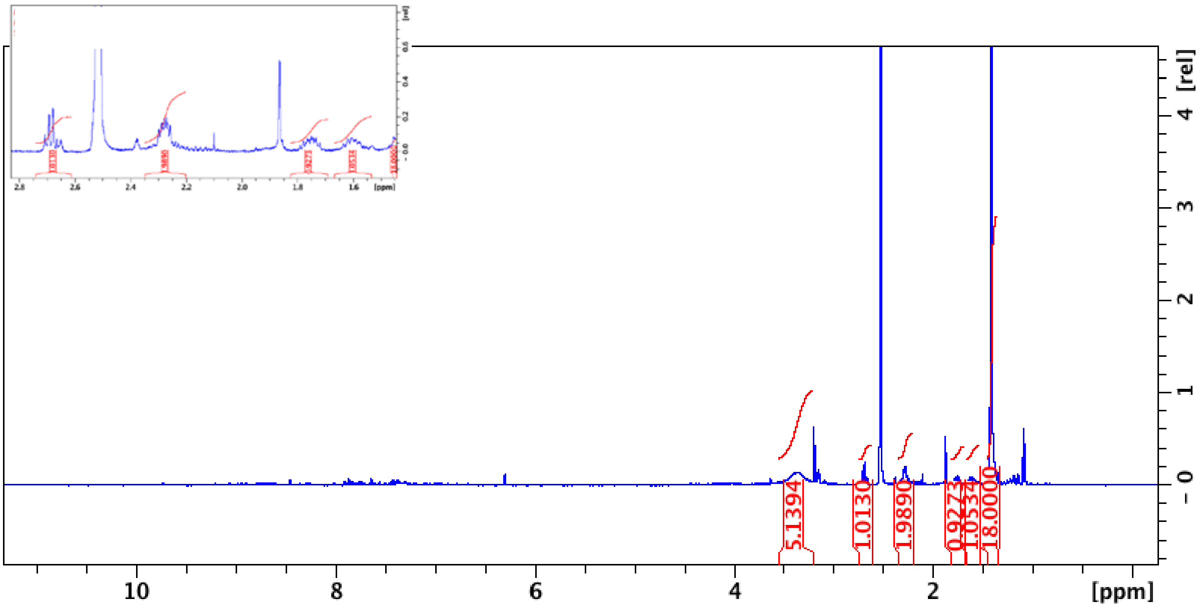


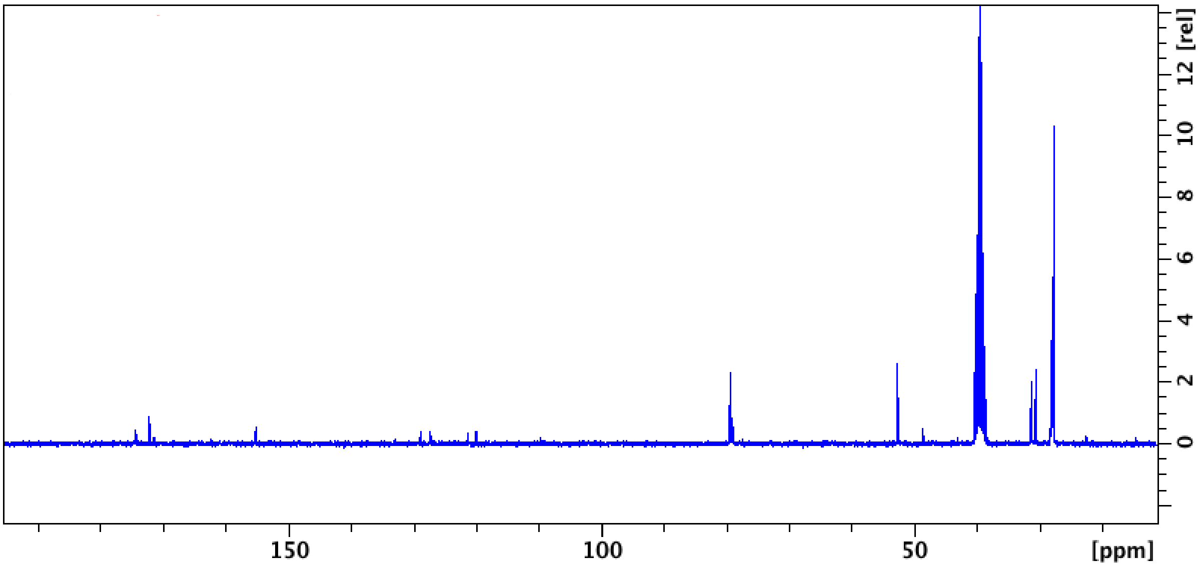

**Ethyl 2-({4-[7,12,17-*tris* ({4-[(2-ethoxy-2-oxoethyl) carbamoyl] phenyl}, )-21,22,23,24-tetraazapentacyclo [16.2.1.1$^{3,6}$.1$^{8,11}$.1$^{13,16}$]tetracosa-1,3,5,7,9,11,13,15,17,19-decaen-2-yl]phenyl}formamido)acetate,**

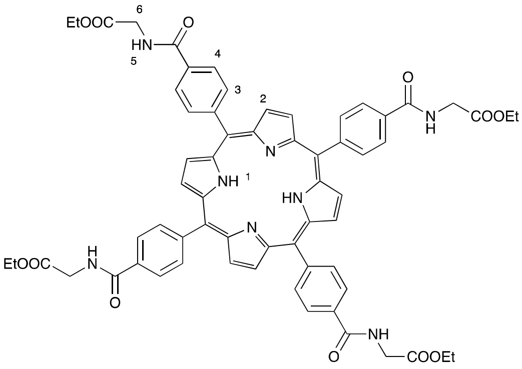


Tetracarboxyphenyl porphyrin (100 mg, 0.126 mmol), PyBOP (394 mg, 0.757 mmol), di*iso*propylethylamine (0.26 mL, 1.51 mmol) and ethyl glycine hydrochloride salt (106 mg, 0.757 mmol) in anhydrous dimethylformamide (5 mL) were stirred for 16 hours. Methyl*iso*cyanate polystyrene resin (0.340g, 0.063 mmol, 200-400 mesh 1.8 mmol/g) was then added and the reaction mixture stirred for a further 3 hours. The solution was then diluted with dichloromethane (50 mL) and washed successively with saturated sodium hydrogen carbonate solution (100 mL), 1 M hydrochloric acid (100 mL) and brine (100 mL). The organic phase was dried (sodium sulfate) and concentrated to yield the crude product as a purple solid. This was purified by flash column chromatography (3:7 ethyl acetate:dichloromethane) to yield the product as a purple solid (45 mg, 0.0397 mmol, 32 %); $^{1}$H NMR (500 MHz, DMSO-$d_6$) δ ppm 1.31 (t, *J* = 6.8 Hz, 12 H, Et $CH_3$), 4.19 (d, *J* = 5.9 Hz, 8 H, H6), 4.24 (q, *J* = 6.8 Hz, 8 H, Et $CH_2$), 8.34 (d, *J* = 8.5 Hz, 8 H, H4), 8.38 (d, *J* = 8.5 Hz, 8 H, H3), 8.82 - 8.94 (s, 4 H, H2), 9.34 (t, *J* = 5.9 Hz, 4 H, H5); IR (solid state, cm$^{-1}$) 3272 (N-H), 1759 (C=O ester), 1638 (C=O amide); ESI-HRMS found *m/z* 1132.4295 [M]$^+$, $[C_{64}H_{60}N_8O_{12}]^+$ requires 1132.4331

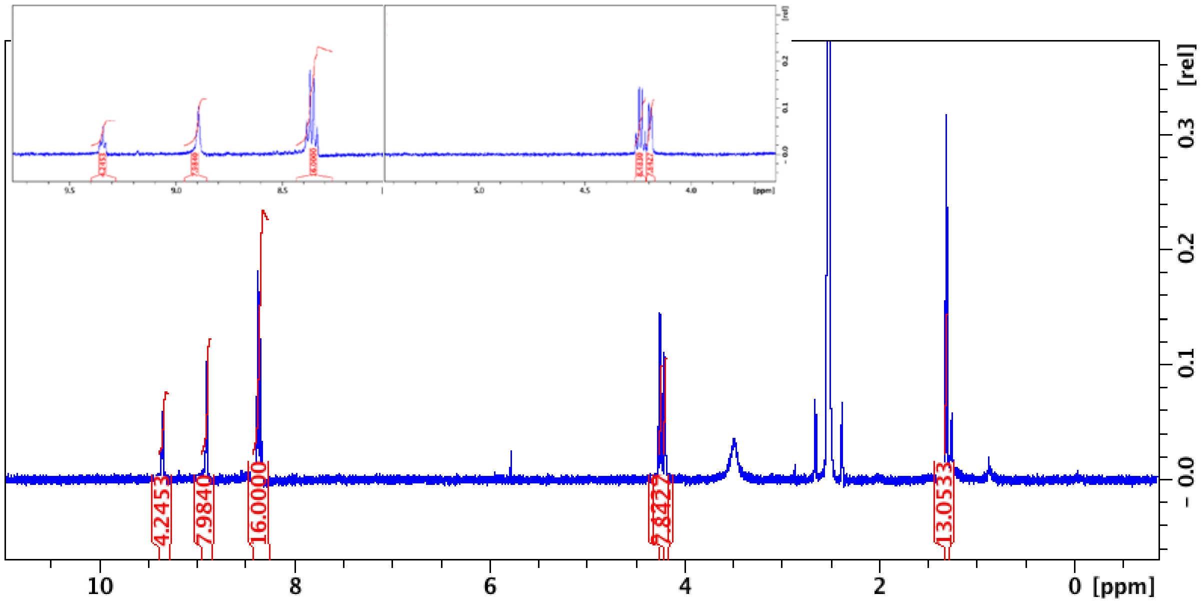

**Methyl (2S)-2-({4-[7,12-*bis*(4-{[(2R)-3-(*tert*-butoxy)-1-methoxy-1-oxopropan-2-yl] carbamoyl} phenyl)-17- (4-{[(2S)-3-(*tert*- butoxy)-1- methoxy- 1-oxopropan-2-yl] carbamoyl} phenyl)-21,22,23,24- tetraazapentacyclo [16.2.1.1³, -⁶.1⁸,¹¹.1¹³,¹⁶] t 1,3 (24), 4, 6, 8, 10, 12, 14, 16 (22), 17,19-undecaen-2-yl] phenyl} formamido)-3-(*tert*-butoxy) propanoate,**

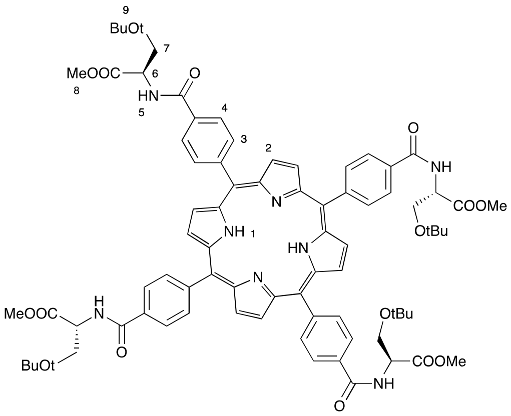

Tetracarboxy phenyl porphyrin (50 mg, 0.067 mmol), PyBOP (197 mg, 0.402 mmol), *O-tert*-butyl-L-serine methyl ester hydrochloride (85 mg, 0.402 mmol), and di*iso*propylethylamine (0.14 mL, 0.40 mmol) in anhydrous dimethylformamide (5 mL) were stirred under a nitrogen atmosphere for 18 hours. The reaction mixture was then dissolved in ethyl acetate (50 mL) and washed with saturated sodium hydrogen carbonate solution (50 mL), 1 M hydrochloric acid (50 mL) and brine (3 × 100 mL). The organic phase was dried (sodium sulfate) and concentrated to yield the product as a purple solid (97 mg, 0.076 mmol, quant.); $^{1}$H NMR (500 MHz, $CDCl_3$) δ ppm 1.18 (s, 36 H, H9), 3.77 - 3.82 (m, 8 H, H8 + H7), 3.98 (dd, *J* = 9.2, 2.7 Hz, 4 H, H7'), 5.05 (dt, *J* = 8.3, 2.7 Hz, 4 H, H6), 7.27 (d, *J* = 8.3 Hz, 4 H, H5), 8.17 (d, *J* = 8.0 Hz, 8 H, H4), 8.22 - 8.29 (d, *J* = 8.0 Hz, 8 H, H3), 8.78 (s, 8 H, H2); $^{13}$C NMR (75 MHz, $CDCl_3$)

δ ppm 27.5, 46.3, 52.6, 62.2, 73.7, 119.4, 125.7, 128.5, 131.2, 133.6, 134.7, 145.5, 167.2, 171.2; IR (solid state, $cm^{-1}$) 3314 (N-H), 1741 (C=O ester), 1656 (C=O amide); ESI-MS *m/z* found 1419.6575 $[M+H]^+$, $[C_{80}H_{90}N_8O_{16}]^+$ requires 1419.6553

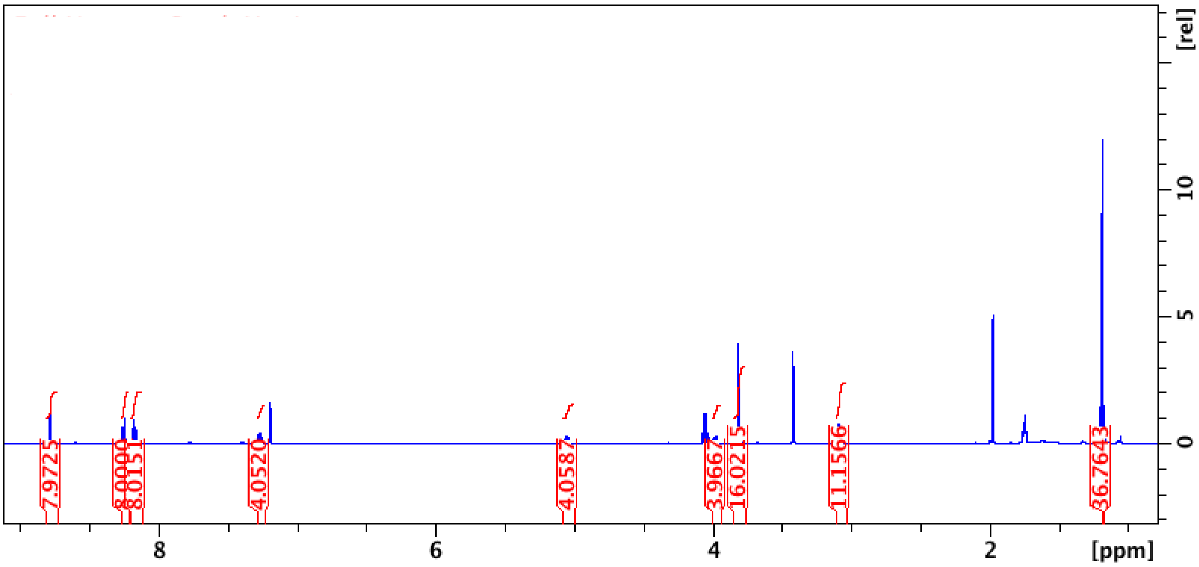


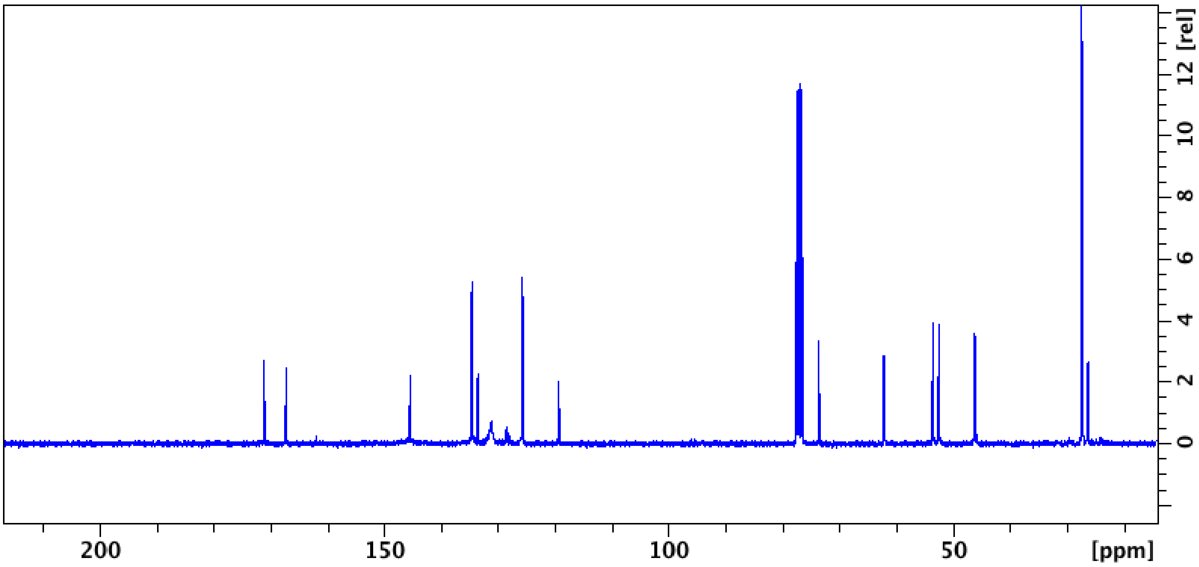

**4-[7,12-bis(4-{[(1R)-2-(*tert*-butoxy)-1-{N'-[(*tert*-butoxy) carbonyl] hydrazine carbonyl} ethyl] carbamoyl} phenyl)-17-(4-{[(1S)-2-(*tert*-butoxy)-1-{N'-[(*tert*-butoxy) carbonyl] hydrazinecarbonyl} ethyl] carbamoyl} phenyl)-21,22,23,24-tetraazapentacyclo [16.2.1.1$^{3,6}$.1$^{8,11}$.1$^{13,16}$] tetracosa-1,3(24), 4,6,8,10,12,14,16(22), 17,19-undecaen-2-yl]-N-[(1S)-2-(*tert*-butoxy)-1-{N'-[(*tert*-butoxy)carbonyl] hydrazine carbonyl} ethyl]benzamide**

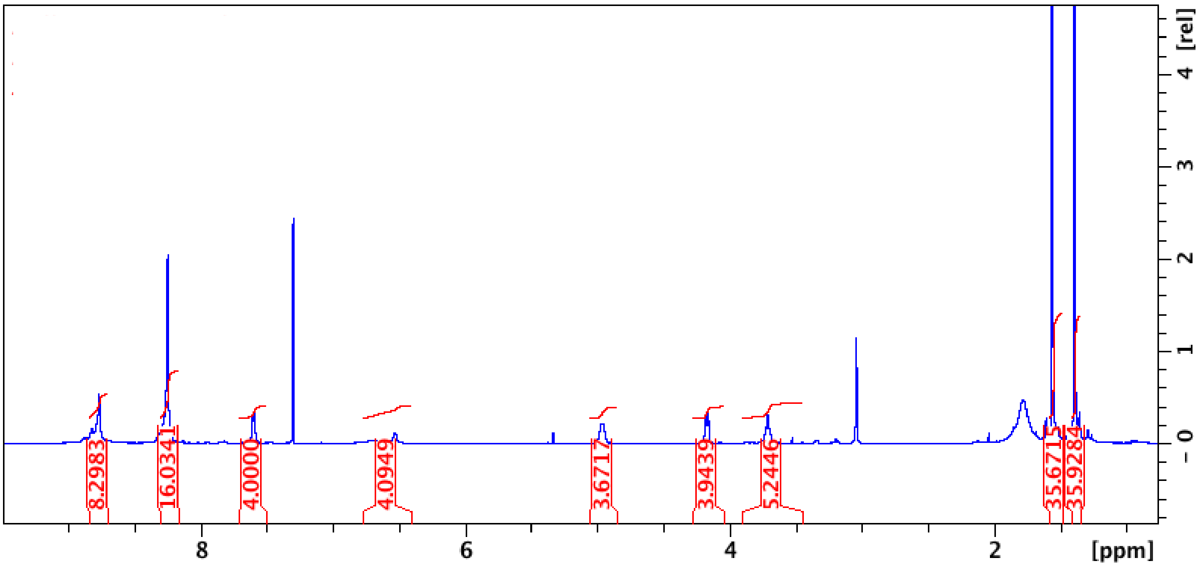


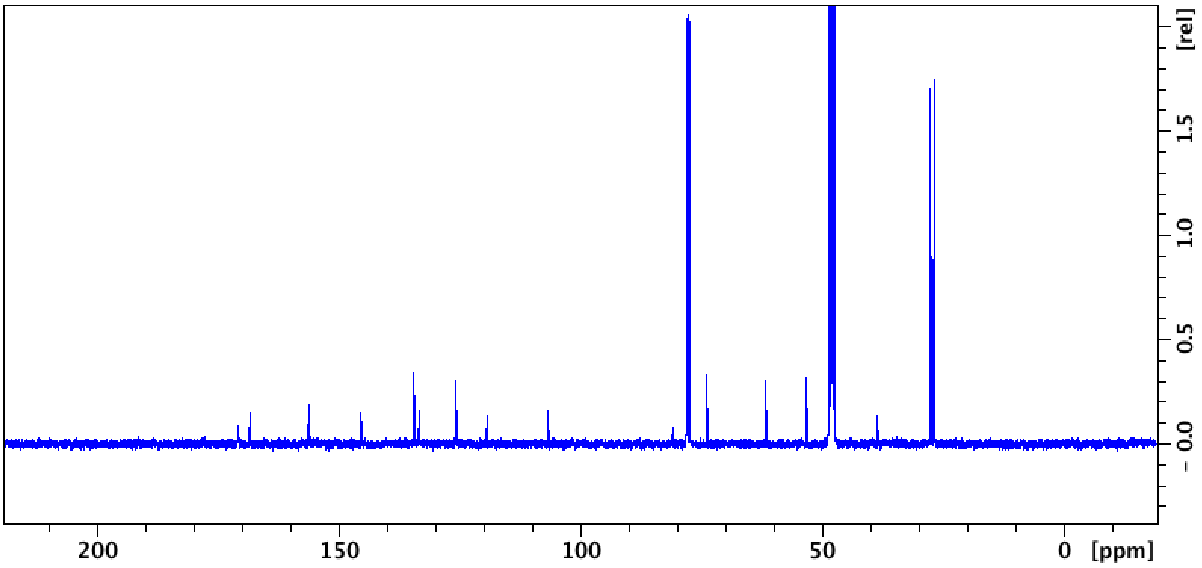

**4-[7,12-bis(4-{[(1R)-2-hydroxy-1-{N'-[(1E)-phenylmethylidene] hydrazinecarbonyl} ethyl] carbamoyl} phenyl)-17-(4-{[(1S)-2-hydroxy-1-{N'-[(1E)-phenylmethylidene] hydrazinecarbonyl} ethyl] carbamoyl} phenyl)-21,22,23,24-tetraazapentacyclo [16.2.1.1$^{3,6}$.1$^{8,11}$.1$^{13,16}$] tetracosa-1,3(24),4,6,8,10,12,14,16(22),17,19-undecaen-2-yl]-N-[(1S)-2-hydroxy-1-{N'-[(1E)-phenylmethylidene] hydrazinecarbonyl}ethyl]benzamide, 1aaaa**

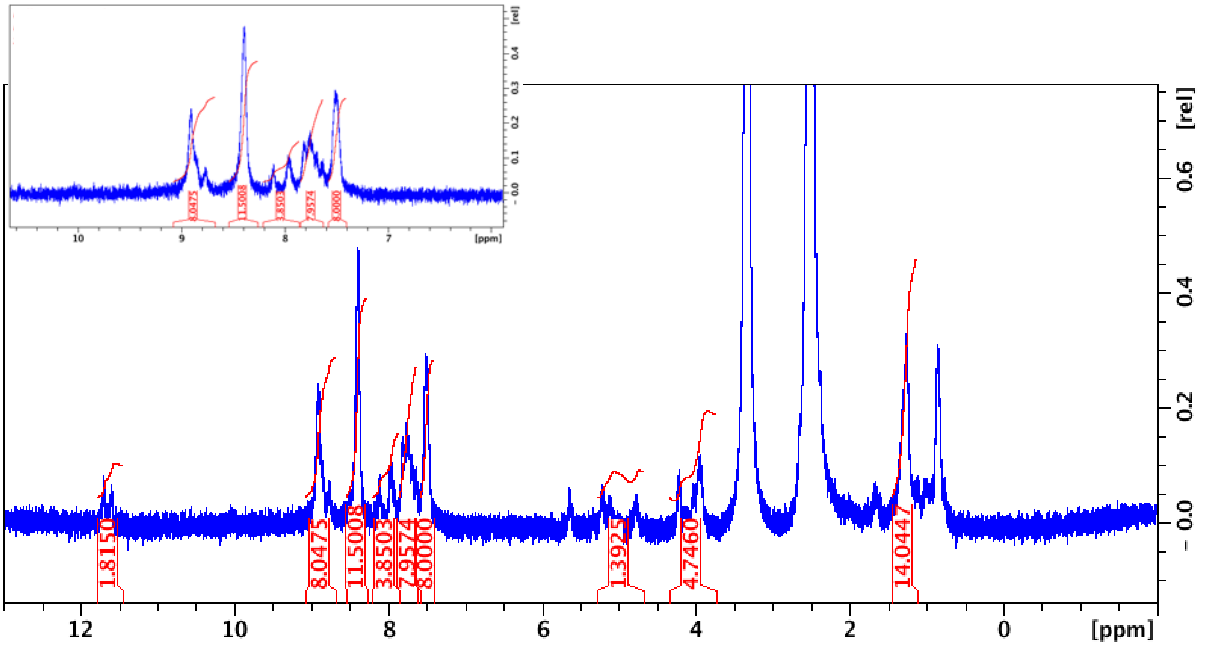

***tert*-butyl (3S)-3-({4-[7,12-bis(4-{[(1R)-3-(*tert*-butoxy)-1-{N'-[(*tert*-butoxy)carbonyl] hydrazinecarbonyl}-3-oxopropyl]carbamoyl}phenyl)-17-(4-{[(1S)-3-(*tert*-butoxy)-1-{N'-[(*tert*-butoxy)carbonyl]hydrazinecarbonyl}-3-oxopropyl]carbamoyl}phenyl)-21,22,23,24-tetraazapentacyclo[16.2.1.$1^{3,6}$.$1^{8,11}$.$1^{13,16}$]tetracosa-1,3(24),4,6,8,10, 12,14,16(22),17,19-undecaen-2-yl]phenyl}formamido)-3-{N'-[(tert-butoxy)carbonyl] hydrazinecarbonyl}propanoate**

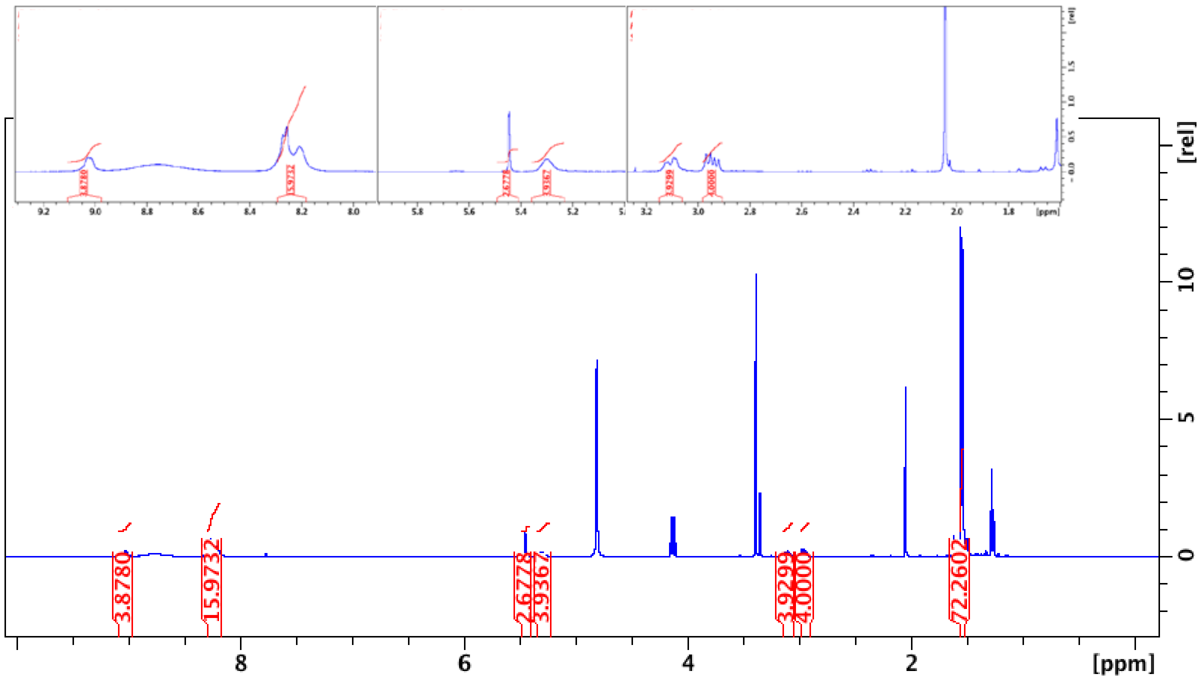


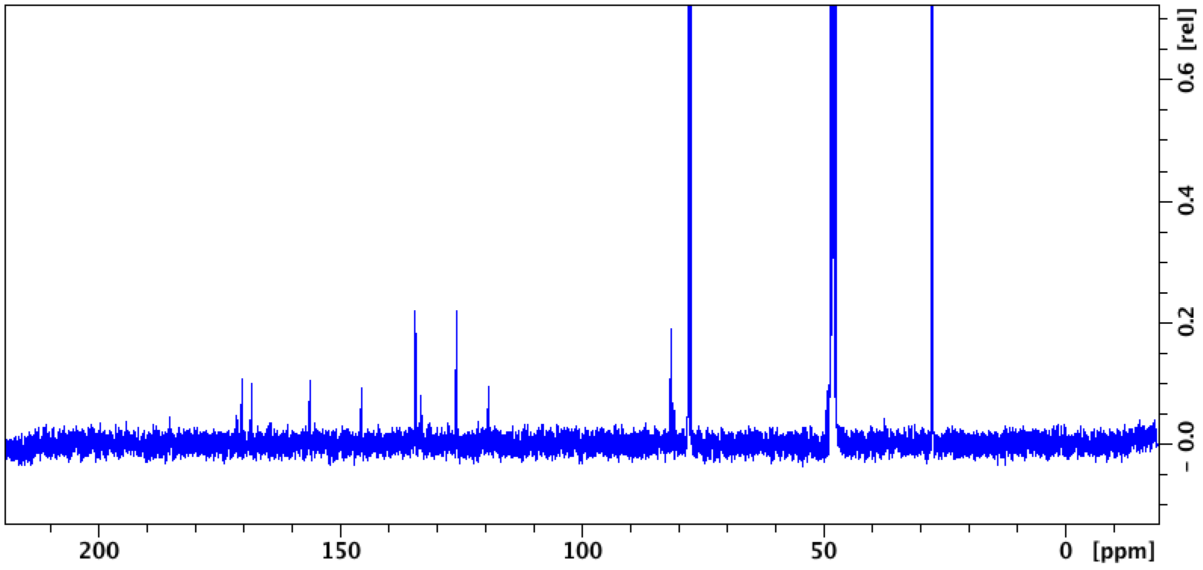

**3S)-3-({4-[7,12-bis(4-{[(1R)-2-carboxy-1-{N'-[(1E)-phenylmethylidene] hydrazinecarbonyl}ethyl]carbamoyl}phenyl)-17-(4-{[(1S)-2-carboxy-1-{N'-[(1E)-phenylmethylidene] hydrazinecarbonyl}ethyl] carbamoyl} phenyl)-21,22,23,24-tetraazapentacyclo[16.2.1.1$^{3,6}$.1$^{8,11}$.1$^{13,16}$]tetracosa-1,3(24),4,6,8,10,12,14,16(22),17,19-undecaen-2-yl]phenyl}formamido)-3-{N'-[(1E)-phenylmethylidene] hydrazinecarbonyl} propanoic acid, 2aaaa**

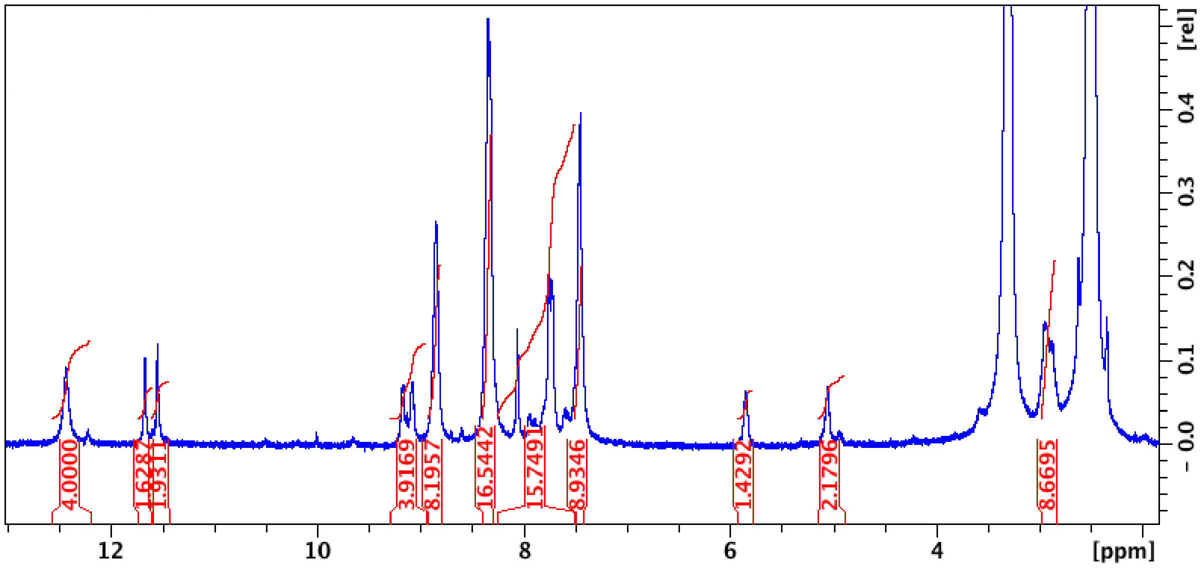

***tert*-butyl (4S)-4-({4-[7,12-bis(4-{[(1R)-4-(*tert*-butoxy)-1-{N'-[(*tert*-butoxy)carbonyl] hydrazinecarbonyl}-4-oxobutyl]carbamoyl}phenyl)-17-(4-{[(1S)-4-(*tert*-butoxy)-1-{N'-[(*tert*-butoxy)carbonyl]hydrazinecarbonyl}-4-oxobutyl] carbamoyl}phenyl)-21,22,23,24-tetraazapentacyclo $[16.2.1.1^{3,6}.1^{8,11}.1^{13,16}]$ tetracosa-1,3(24),4,6,8,10,12,14,16(22),17,19-undecaen-2-yl] phenyl} formamido)-4-{N'-[(*tert*-butoxy)carbonyl] hydrazine carbonyl} butanoate**

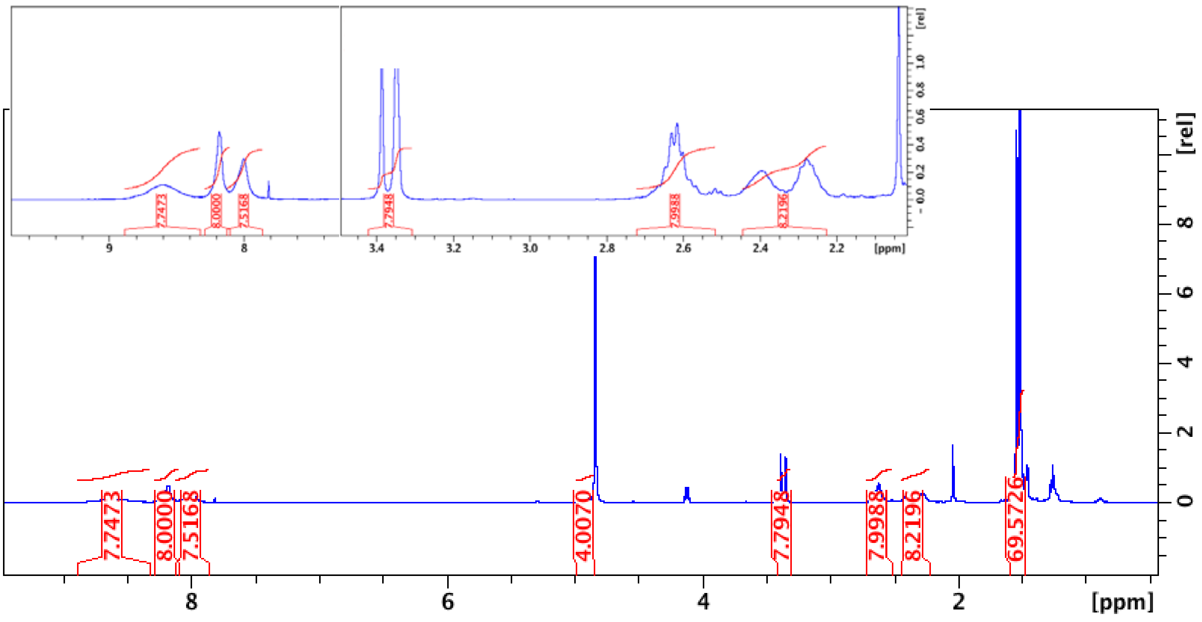


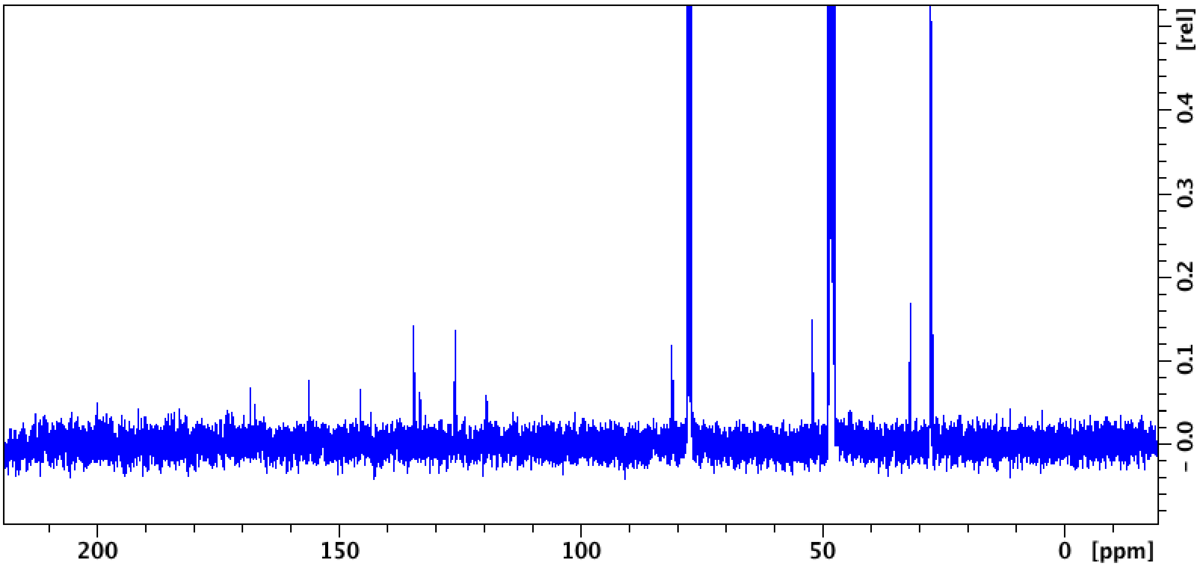


**(4S)-4-({4-[7,12-bis(4-{[(1R)-3-carboxy-1-{N'-[(1E)-phenylmethylidene]hydrazine carbonyl}propyl]carbamoyl}phenyl)-17-(4-{[(1S)-3-carboxy-1-{N'-[(1E)-phenyl**

**methylidene]hydrazinecarbonyl}propyl]carbamoyl}phenyl)-21,22,23,24-tetraaza pentacyclo[16.2.1.$1^{3,6}$.$1^{8,11}$.$1^{13,16}$]tetracosa-1,3(24),4,6,8,10,12,14,16(22),17,19-undecaen-2-yl]phenyl}formamido)-4-{N'-[(1E)-phenylmethylidene]hydrazinecarbonyl} butanoic acid, 3aaaa**

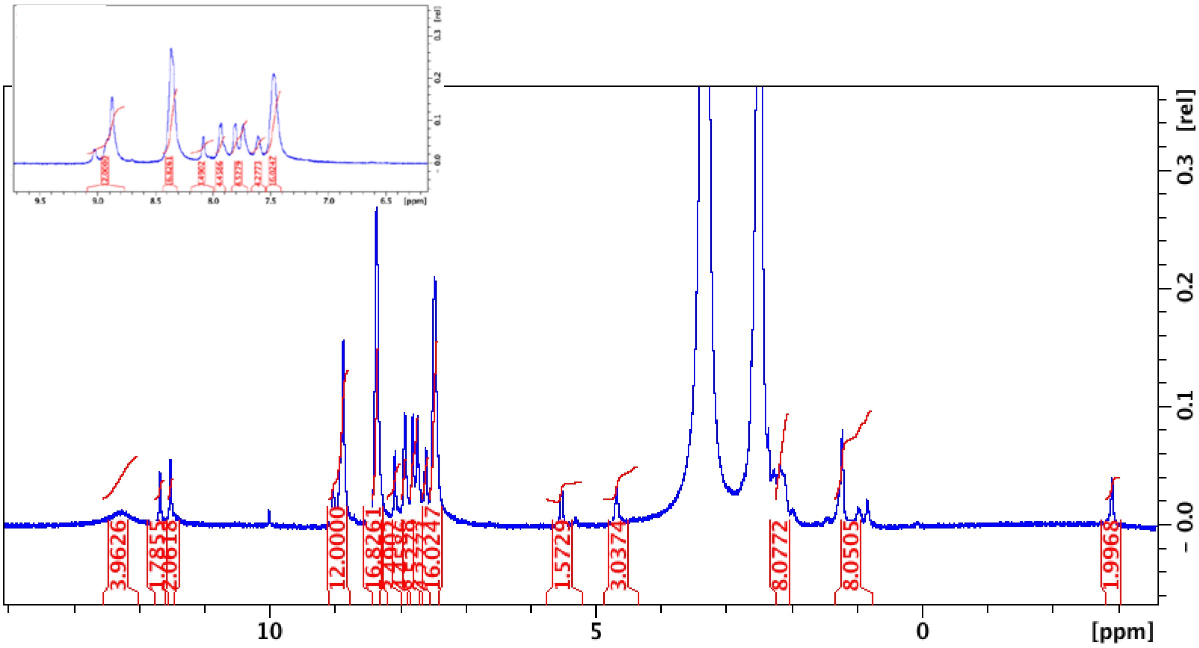


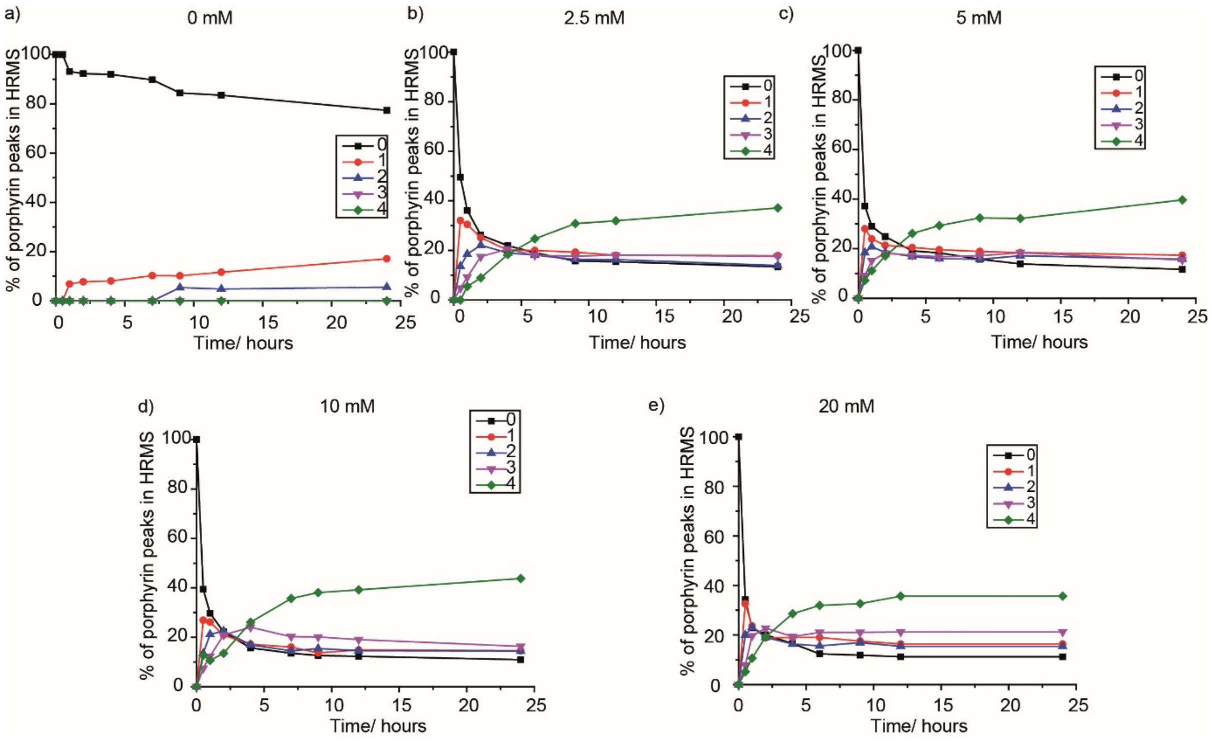

**Figure ESI 1**. Effect of catalyst concentration on hydrazone exchange for aspartic acid hydrazone porphyrin scaffold **2aaaa** (100 μM) with 2,4-dimethoxy benzaldehyde **10** (5 mM) and varying concentrations of aniline **11** (10 % DMSO in 5 mM $NH_4OAc$, pH 6.75)

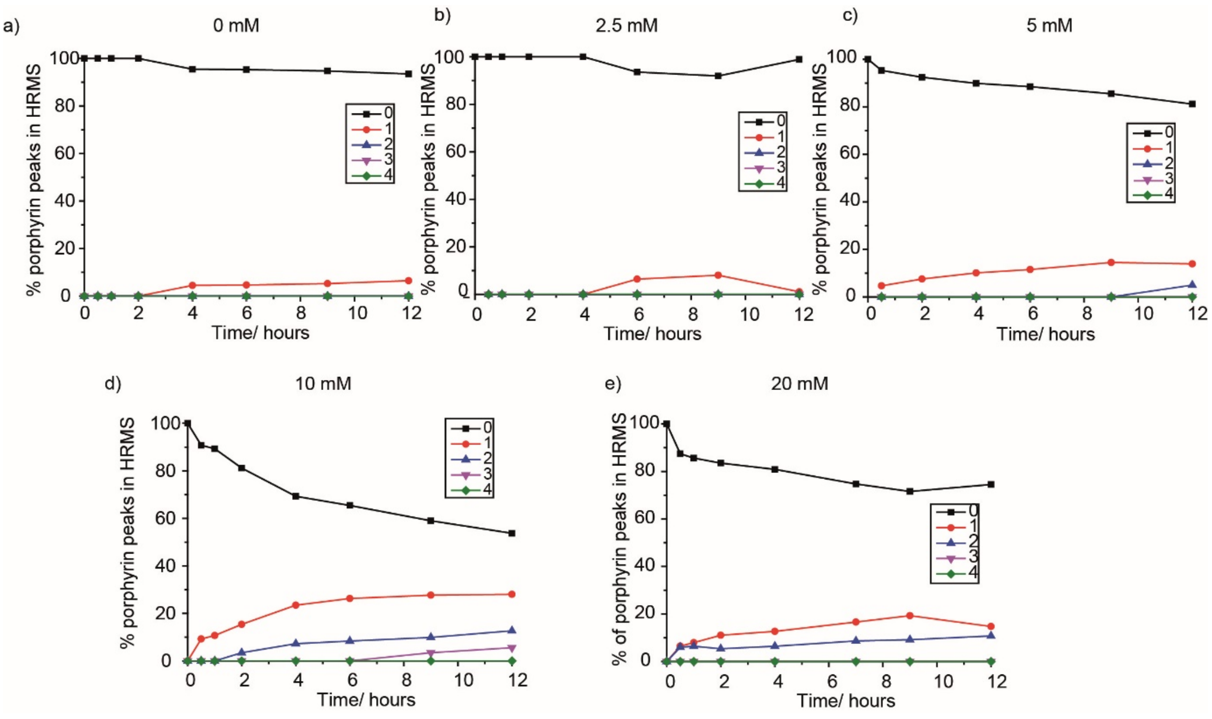


**Figure ESI 2**. Effect of catalyst concentration on hydrazone exchange for serine hydrazone porphyrin scaffold **1aaaa** (100 μM) with 2,4-dimethoxy benzaldehyde **10** (5 mM) and varying concentrations of aniline **11** (10 % DMSO in 5 mM $NH_4OAc$, pH 6.75)

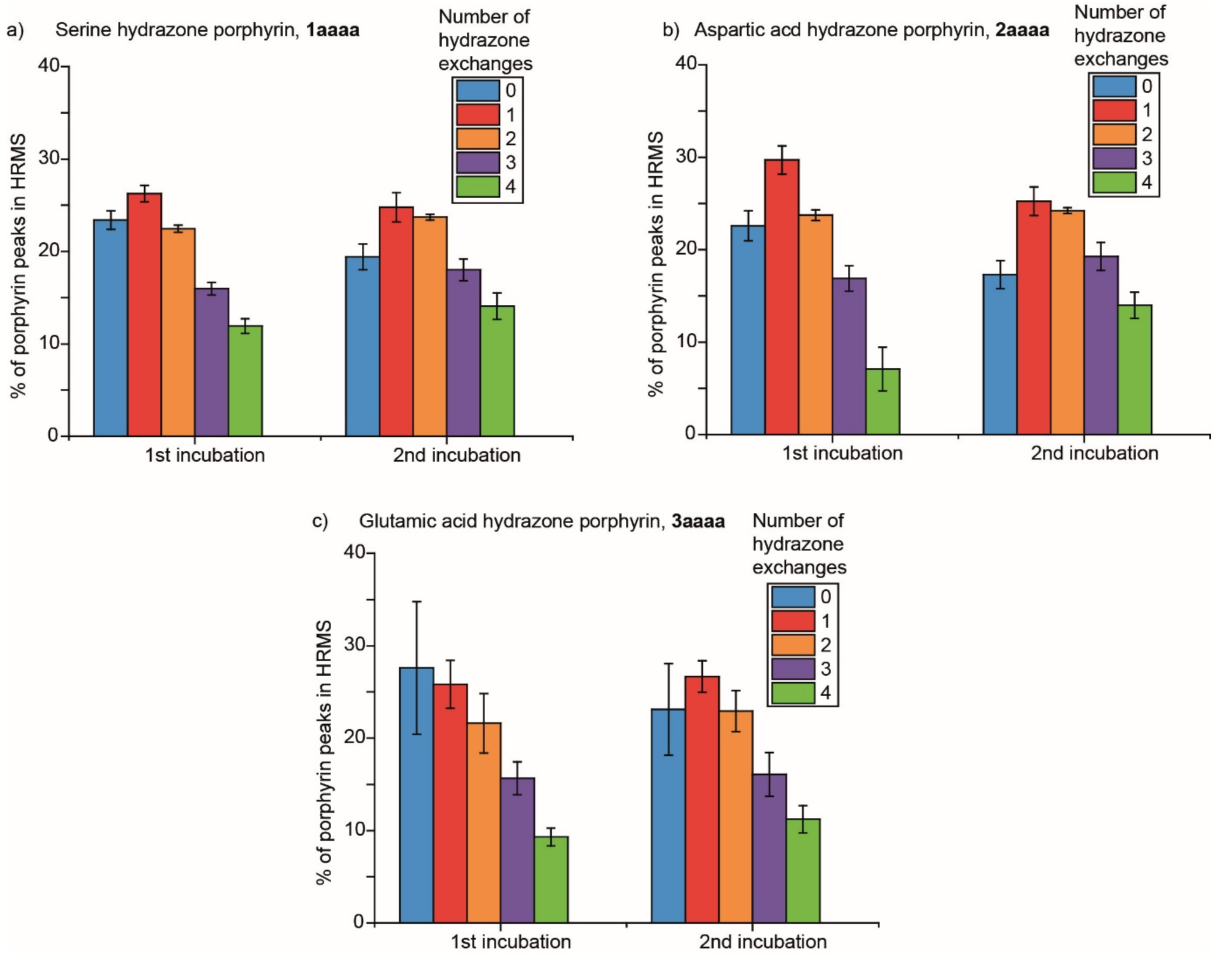


**Figure ESI 3.** Addition of 2 separate batches of aldehyde to probe re-equilibriation: incubation with 25 eq. of aldehyde **10** for 24 hours followed by addition of a second 25 eq. of aldehyde **10**. First incubation of 100 μM hydrazone porphyrin **1-3aaaa** with 2.5 mM 4-carboxy benzaldehyde **10** and 10 mM aniline **11** (10 % DMSO in 5 mM $NH_4OAc$, pH 6.75). Second incubation, addition of a further 2.5 mM 4-carboxy benzaldehyde 10 to first incubation. Graphs show an average of 5 separate measurements (a) serine hydrazone porphyrin **1aaaa**, (b) aspartic acid hydrazone porphyrin **2aaaa**, (c) glutamic acid hydrazone porphyrin **3aaaa**.
